# Uncovering Diversity within the Glomeromycota: Novel Clades, Family Distributions, and Land Use Sensitivity

**DOI:** 10.1002/ece3.70597

**Published:** 2025-01-08

**Authors:** Camille S. Delavaux, Alexis Aellen, Sidney L. Stürmer, Silmar Primieri, Ursel M. E. Schütte, Devin M. Drown, Robert J. Ramos, Thomas W. Crowther, James D. Bever

**Affiliations:** ^1^ Institute of Integrative Biology ETH Zurich (Swiss Federal Institute of Technology) Zurich Switzerland; ^2^ Department of Ecology and Evolutionary Biology The University of Kansas Lawrence Kansas USA; ^3^ Departamento de Ciências Naturais Universidade Regional de Blumenau Blumenau Santa Catarina Brazil; ^4^ Instituto Federal de Santa Catarina (IFSC) Lages Santa Catarina Brazil; ^5^ Institute of Arctic Biology University of Alaska Fairbanks Fairbanks Alaska USA; ^6^ Department of Biology and Wildlife University of Alaska Fairbanks Fairbanks Alaska USA; ^7^ Kansas Biological Survey The University of Kansas Lawrence Kansas USA; ^8^ The Environmental Data Science Innovation & Inclusion lab (ESIIL) at the University of Colorado Boulder Boulder Colorado USA

**Keywords:** biogeography, Glomeromycota, land use history, large subunit rDNA, phylogenetics

## Abstract

Arbuscular mycorrhizal fungi (AMF, phylum Glomeromycota) are essential to plant community diversity and ecosystem functioning. However, increasing human land use represents a major threat to native AMF globally. Characterizing the loss of AMF diversity remains challenging because many taxa are undescribed, resulting in poor documentation of their biogeography and family‐level disturbance sensitivity. We survey sites representing native and human‐altered ecosystems across the American continents—in Alaska, Kansas, and Brazil—to shed light on these gaps. Using a recently developed pipeline for phylogenetic placement of eDNA, we find evidence for three putative novel clades within the Glomeromycota, sister to *Entrophosporaceae*, *Glomeraceae*, and *Archaeosporaceae*, with evidence for geographic structuring. We further find that taxa in the *Diversisporaceae*, *Glomeraceae*, and *Entrophosporaceae* relatively high families are overrepresented and more diverse in temperate samples. By contrast, the diversity of taxa that cannot be placed into a family is higher in tropical samples, suggesting that tropical sites harbor relatively high undescribed AMF diversity. Moreover, we find evidence that *Entrophosporaceae* is more tolerant, while *Glomeraceae* is more sensitive to disturbance. These results underscore the vast undescribed diversity of AMF while highlighting a way forward to systematically improve our understanding of AMF biogeography and response to human disturbance.

## Graphical abstract

Using a recently developed pipeline for phylogenetic placement of arbuscular mycorrhizal fungi (AMF) with samples across sites in Alaska, Kansas, and Brazil, we find evidence for three putative novel clades within the Glomeromycota, sister to Entrophosporaceae, Glomeraceae, and Archaeosporaceae. Moreover, we find evidence that Entrophosporaceae is more tolerant, while Glomeraceae is more sensitive to disturbance. These results underscore the vast undescribed diversity of AMF while highlighting a way forward to systematically improve our understanding of AMF biogeography and response to human disturbance.

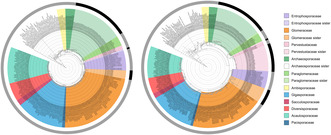

## 1 Introduction

Arbuscular mycorrhizal fungi (AMF, phylum Glomeromycota) are ubiquitous symbionts that associate with over 70% of terrestrial plant species (Brundrett and Tedersoo 2018), providing both nutritional and non‐nutritional benefits to host plants (Delavaux, Smith‐Ramesh, and Kuebbing 2017). Despite the ubiquity and importance of these fungi across the globe, our understanding of their diversity and distribution is limited. Unfortunately, many AMF taxa remain undescribed—particularly in undersampled regions—and may be lost before we discover them (Chaudhary, Lau, and Johnson 2008). Uncovering the complete diversity of AMF is essential to robustly answer pressing questions relevant to mycorrhizal ecology, plant community ecology, and restoration ecology. Major unanswered questions remain regarding AMF distributions across bioclimatic zones, level of endemism, and response to ever‐growing anthropogenic forces (Stürmer, Bever, and Morton 2018; Davison et al. 2015; Cotton 2018; Johnson et al. 2013). As such, there is an urgent need to develop and validate methods to enable a description of AMF diversity.

Characterizing the total extent of AMF diversity remains challenging due, in part, to the lack of a systematic method to identify novel, previously unknown taxa. Because AMF are obligate symbionts, they cannot be cultured via plating like many other fungi (Smith and Read 2008; Varma and Hock 2013). Instead, these fungi must be cultured with host plants, requiring large greenhouse and laboratory facilities alongside constant maintenance. Besides being extremely resource and time‐intensive, this practice is biased by plant host choice. Most culturing of AMF is done using corn ( *Zea mays* ) or sorghum ( *Sorghum bicolor* ; https://invam.ku.edu/), which likely filters which fungi can be successfully cultured. This is because AMF exhibit specificity in response to and impact on host plants, resulting in each host plant species having a different probability to culture a given AMF taxa (Bever et al. 1996; Mangan, Herre, and Bever 2010; Cheeke et al. 2019). The majority of culturing and traditional taxonomic placement (i.e., through spore morphology) of these fungi has been conducted overwhelmingly in temperate regions (although there is a concerted effort in additional regions, e.g., South America), further skewing our understanding of this diverse group (Stürmer, Bever, and Morton 2018).

In the last two decades, these traditional approaches have been complemented by environmental sequencing, which has broadened our understanding of the distribution patterns of this group (Stockinger, Krüger, and Schüssle 2010). However, the most common application of environmental short read (Illumina) sequencing (Tedersoo et al. 2022; Hart et al. 2015; Kolaříková et al. 2021) relies on geographically and taxonomically restricted databases, sometimes disregarding taxa that do not have matches in these databases (Hart et al. 2015), limiting taxonomic information and the discovery of novel taxa. To more thoroughly assess AMF diversity—including unknown, uncultured, and unculturable AMF—recent work has developed a pipeline focused on the large subunit (LSU) region of rDNA that enables phylogenetic placement of any environmental sequence (Delavaux, Sturmer, et al. 2021). The reference backbone tree was built by integrating information from taxa at the genus and family level described to date. Further refinement of this pipeline additionally delineated the major AMF families for putative assignment of environmental sequences (Delavaux et al. 2022; Delavaux, Ramos, et al. 2024). Importantly, the phylogenetic placement of these taxa now enables a better understanding of AMF diversity and taxonomy, allowing for expansion of existing families or even discovery of novel clades through environmental sampling.

Relatively little work has assessed the changing representation of AMF families across latitudinal gradients, across the major bioclimatic zones from boreal to tropical. Focusing on family‐level patterns is particularly important as traits are known to be conserved at this level (Hart and Reader 2002; Maherali and Klironomos 2012). A handful of studies has assessed the biogeography of AMF (Davison et al. 2015; Öpik et al. 2010), but either has not split the results by family or reports only a limited number of families. Additional research has focused on describing the niches of AMF families (Vasar et al. 2022; Veresoglou, Caruso, and Rillig 2013; Davison et al. 2021), but does not directly compare prevalence across bioclimatic zones. To date, the most relevant AMF biogeography study came from a compilation of studies including both molecular and morphological identification of AMF species (Stürmer, Bever, and Morton 2018), suggesting that AMF from the families *Diversisporaceae*, *Glomeraceae*, and *Paraglomeraceae* are overrepresented in boreal systems, AMF from the families *Glomeraceae* and *Paraglomeraceae* are overrepresented in temperate systems, and AMF from the families *Acaulosporaceae*, *Gigasporaceae*, and *Ambisporaceae* are overrepresented in tropical systems. However, whether these patterns hold with consistent field sampling and data analyses remains unclear. Given recent advancements in phylogenetic placement tools for AMF, the time is ripe to sample across a broader geographic range to uncover novel groups within the Glomeromycota and refine our understanding of detailed family‐level biogeographic distributions.

Like most aspects of biodiversity, AMF diversity is directly impacted by anthropogenic land use change, including the conversion of natural ecosystems to agriculture and subsequent abandonment of this land. Recent evidence shows that agriculture degrades the AMF community in particular, altering diversity and community composition (House and Bever 2018; Tipton et al. 2019; Roy et al. 2017). In fact, restoration of plant communities, especially of desirable late successional species, has been shown to depend on the reintroduction of native AMF (Koziol and Bever 2016; Koziol, Mckenna, and Bever 2023; Koziol et al. 2023, 2018). As humans convert land, host plants are removed, and conditions may no longer be beneficial to the partnership. AMF are dispersal limited (Tipton et al. 2022; Delavaux, Weigelt, et al. 2021; Chaudhary et al. 2020; García De León et al. 2016), making their reintroduction exceedingly slow, if not improbable. Most restoration approaches apply a mix of diverse AMF; however, due to the difficulty of culturing and generating inocula, reintroduction of especially degraded components (e.g., families) of the mycorrhizal community could make restorations more cost effective and targeted. Understanding the impact of land use—including conversion to agriculture, and subsequent abandonment as post‐agricultural lands—will be essential to determine which taxa are lost from these systems. This is not only useful in understanding how these systems may recover, but importantly, how we might reverse this loss by reintroduction of particularly impacted groups for successful restoration. Therefore, more detailed analysis of which AMF families are particularly impacted by human land use should help improve targeted restoration efforts on the ground.

An understanding of which AMF families are most sensitive or resistant to land use change is beginning to emerge. Chagnon et al. (2013) described a competition–stress tolerant–ruderal (CSR) framework, adapting Grime's triangle (Grime 2006) to apply a life history classification to the three major AMF families. Further research has expanded our expectations by explicitly testing the over and under representation of specific families in response to disturbance. Based on this previous work (Cahyaningtyas and Ezawa 2023; Chen et al. 2023; Chagnon et al. 2013; Oehl et al. 2003; Van Der Heyde et al. 2017; Kajihara et al. 2022; House and Bever 2018; Tipton et al. 2019; Větrovský et al. 2023), we expect that taxa in the *Acaulosporaceae*, *Archeosporacae*, and *Gigasporaceae* families are disturbance sensitive, while taxa in the *Diversisporaceae*, *Entrophosporaceae* (formerly *Claroideoglomeraceae*), *Pacisporaceae*, and *Sacculosporaceae* families are disturbance tolerant. Taxa in the *Glomeraceae* have been found in relatively equal instances to be either tolerant (Van Der Heyde et al. 2017; Chagnon et al. 2013; Cahyaningtyas and Ezawa 2023) or sensitive (Chen et al. 2023; House and Bever 2018). This may not be surprising given how large and diverse this family is (Błaszkowski et al. 2023). In addition, it is important to note that most studies testing disturbance impacts on AMF families have been conducted in temperate regions, meaning that our current understanding is further biased toward these regions. Therefore, to refine our understanding of tolerant versus sensitive AMF families, it is crucial that we incorporate a more diverse range of bioclimatic zones.

Here, we leverage the previously published LSU AMF database and pipeline (Delavaux et al. 2022; Delavaux, Ramos, et al. 2024; Delavaux, Sturmer, et al. 2021) alongside sampling across land use in ecosystems from three bioclimatic zones—boreal, temperate, and tropical—to (1) uncover new AMF diversity (2) refine our understanding of the biogeography of AMF families and (3) understand how land use impacts diversity and local proportions of AMF families. This enables an assessment of the potential for the phylogenetic placement method to uncover novel AMF clades, identify biogeographical patterns at the family level, and further examine effects of land‐use change on these essential fungi. Even with our relatively limited sampling effort across three bioclimatic zones, we find support for three novel clades within the Glomeromycota. Additionally, we identify previously uncatalogued family level biogeographic patterns. Moreover, we report on both consistent and bioclimatic zone‐specific effects of land use on particular AMF families. Thus, this work serves as a validation of this pipeline's potential to identify previously overlooked AMF diversity, reexamine our view of AMF biogeography, and determine AMF families of potential restoration focus.

## 2 Materials and Methods

### 2.1 Sampling Design

Sampling took place in 2017 in Alaska (boreal bioclimatic zone) and in Brazil (tropical bioclimatic zone), and in 2018 and 2019 in Kansas (temperate bioclimatic zone). Land use of sites was classified as either remnant (native, without obvious human disturbance), agricultural (actively used for agriculture), or post‐agricultural (abandoned agricultural land). Details on current agricultural uses (for agricultural sites) are documented in Table S1. At each site, soil samples were obtained from the top 10–20 cm of soil (Brazil = 20 cm, Alaska = variable). In total, 55 sites and 529 samples were analyzed. In Kansas, soil samples were obtained at six depths (5, 15, 30, 75, 120, and 150 cm). We included all depths when building phylogenetic trees to search for novel AMF clades (all 529 samples), but otherwise only included the most shallow depth of the 2019 sampling for comparison with the other two sites (total 102 samples). DNA extraction and sequencing of samples was carried out Genomic Sequencing Core at the University of Kansas.

### 2.2 DNA Extraction and Bioinformatics

DNA was extracted from soil samples using the Qiagen PowerSoil Kit (Hilden, Germany). As described in Delavaux, Sturmer, et al. (2021), a two‐step PCR consisting of a first PCR and a second PCR to attach indices was conducted using the primer pair LROR‐FLR2 for 2 × 300 MiSeq sequencing. Total sequencing depth was 14,274,866 reads. The pipeline described in Delavaux et al. (2022), Delavaux, Ramos, et al. (2024) to phylogenetically place all amplicon sequence variants (ASVs) into the backbone tree was used. In brief, the associated database and backbone tree were derived using full‐length LSU reads from the International Culture Collection of (Vesicular) Arbuscular Mycorrhizal Fungi (INVAM), the National Center for Biotechnology Information (NCBI), and Krüger et al. (2012). The pipeline then cleans, trims and quality filters all ASVs, merges forward and reverse reads, runs a pre‐BLAST against the database to remove non‐homologous sequences (sequences that do not show any match to reference sequences; 20,263 ASVs), and finally, aligns and places ASVs on the backbone tree in small batches. The pipeline extracts ASVs that fall within the Glomeromycota AMF clade to define these as putative AMF; it also extracts ASVs that fall within the 11 major families. We use this database and pipeline here to identify putative AMF broadly (hereafter AMF), AMF belonging to each family, and AMF that are outside of families represented in the backbone tree, but still within the Glomeromycota AMF clade (unknown). This resulted in 2540 ASVs assigned as AMF.

To enable a comparison of our results to those that would be obtained using a traditional Basic Local Alignment Search Tool (BLAST) approach, we ran BLASTn (blast.ncbi.nlm.nih.gov) on the same ASVs used to build phylogenetic trees for putative AMF placement (i.e., same starting list of ASVs for both methods). Specifically, we matched these sequences against the full Glomeromycota database extracted from the NCBI. The keyword “Glomeromycota” was used to subset all NCBI entries for this phylum (extracted April 6, 2023). From the results of this matching, we retained the top hit by bit score; we subsequently assigned all ASVs that had a percent identity score lower than 98% to non‐AMF. This resulted in 2613 AMF assigned ASVs with this approach. Finally, we used the Entrez‐direct software (Kans 2024) to extract taxonomic information from the NCBI hit IDs, and sorted the ASVs into AMF family. ASVs that matched with Glomeromycota sp. were labeled as unknown. Together, this resulted in one dataset for the (1) tree placement method and one for the (2) BLAST database matching method, where each ASV was classified into one of the 11 families or labeled as unknown (AMF, but without a designated family).

### 2.3 Analyses

Our analyses encompassed two main goals. The first was to identify potential novel AMF families (clades). We define novel clades as groups of ASVs that are within the AMF clade and cluster together, but are separated from known families. To do this, we constructed phylogenetic trees placing ASVs that were not able to be placed in a family (‘unknown’ AMF taxa; still within the Glomeromycota). Our second goal was to understand how AMF diversity and proportion varied by bioclimatic zone, AMF family and land use. We therefore built models predicting either diversity metrics or proportion of each family from bioclimatic zones and land use. Subsequent differential abundance analyses were carried out to investigate within family ASV variation across land use. All analyses were done in R 3.4.1 (Team 2019), with linear mixed models constructed with the lme4 (Bates et al. 2015) and lmerTest (Kunzetsova, Brockhoff, and Christensen 2017) or glmTMBB (Brooks et al. 2023) packages and differential abundance tested with DESeq2 (Love, Anders, and Huber 2014). Additional packages used for data preparation and visualization include tidyverse (Wickham et al. 2019), phyloseq (McMurdie and Holmes 2013), emmeans (Lenth et al. 2019), car (Fox et al. 2012), maps (Brownrigg 2013), sf (Pebesma and Bivand 2023), vegan (Oksanen et al. 2013), DHARMa (Hartig and Hartig 2017), viridis (Garnier et al. 2018), RColorBrewer (Neuwirth and Neuwirth 2014), and cowplot (Wilke, Wickham, and Wilke 2019). Verification of model assumptions was conducted on all models using code from Zuur et al. (2009).

### 2.4 Constructing Phylogenetic Trees to Place Unknown AMF Taxa

We constructed two phylogenetic trees to identify where the taxa that were classified as Glomeromycota but not assigned to family (unknown) may be placed within the phylogeny. The first tree placed only taxa that were unknown to both the tree placement and BLAST approach (‘double unknown’), as these are unknown to both methods. These represent taxa that neither the traditional BLAST approach nor our tree‐based phylogenetic method could place into family. The second tree placed taxa that were unknown to only the tree placement approach (‘tree unknown’), as this is likely the most precise and robust placement into family (Delavaux, Sturmer, et al. 2021; Delavaux et al. 2022) (see Section 3.1 for details regarding BLAST misclassification). These two groups of unknown ASVs were placed into the reference backbone tree using RaxML 8.2.12 (Stamatakis 2014) with 1000 bootstrap replicates and the evolutionary model GTRGAMMA.

### 2.5 Effects of Bioclimatic Zone and Land Use on Family‐Level Diversity and Sequence Read Proportions

To understand the drivers of AMF diversity and proportion of taxa, we tested the impact of family, bioclimatic zone, and land use on (1) three metrics of diversity (observed ASV richness, Shannon diversity, and Chao1 diversity) and (2) the proportion of sequence reads (after subsetting all reads belonging to a given family). We ran all models using either (1) the two land use classifications (remnant versus disturbed, including agricultural and post‐agricultural) across all three bioclimatic zones or (2) the three land use classification (remnant, agricultural, and post‐agricultural) across the temperate and tropical bioclimatic zones. All two category models were run separately using data from the tree placement method and data from BLAST family assignments. Subsequent three category models were only run for data from the tree placement method, as this was considered the more robust assignment. Contrasts were obtained through manually coding pairwise comparisons of model coefficients with least squares means using the emmeans package (Lenth et al. 2019). Throughout the text, we report statistical results for metrics derived from the tree placement method. All contrast results can be found in Tables S2 (Diversity) and S3 (Proportion). Full model code can be found on the study Github repository (https://github.com/c383d893/DiversityofGlomeromycota, [dataset]).

For the diversity models, we used generalized linear mixed models (GLMMs). Each diversity metric had three associated models. This included a ‘full’ model, and two ‘restricted’ models. For the full model, fixed effects were bioclimatic zone, AMF family, all pairwise interactions between bioclimatic zone, land use, and AMF family, and the three‐way interaction between bioclimatic zone, land use, and AMF family. We also included the random effect of replicate nested within bioclimatic zone:state:site to account for non‐independence of observations. All observed richness models contained sample read number as a covariate to account for differential read depth across samples. Because these models did not comply with model assumptions and/or failed to converge, we ran restricted models with either negative binomial (observed richness) or tweedie distributions (Shannon and Chao1 diversity), in particular to correct for high numbers of zeros in our dataset. To run these models with convergence, we removed families that consisted overwhelmingly of zeros in sequence; that is, we removed high zero families (> 70% zeros) one by one until the model would converge. We also removed the boreal bioclimatic zone due to high prevalence of samples with zero values. Because running the three‐way interaction of the restricted models would not converge, we ran two such models: one model including the interaction of AMF family by bioclimatic zone and another, including the interaction of AMF family by land use. In addition, these restricted models included random effects (as described for the full model above; except the BLAST Shannon diversity model). This tiered approach allowed us to robustly statistically test differences across AMF families (for included families) between temperate and tropical bioclimatic zones as well as between remnant and disturbed land use categories. All presented plots include estimated means from the full model (with increased transparency) and estimated means and standard errors from the more restricted models that comply with statistical assumptions.

We modeled the proportion of sequence reads again using GLMMs. We used the same full versus restricted approach described above for these models. However, we were able to run the three‐way interaction in the restricted models, allowing for expanded contrasts, including an interaction between land use and bioclimatic zone. This allowed us to test whether differences in proportion of reads between land uses varied between temperate and tropical bioclimatic zones. We used a binomial distribution, as we used a combined response variable comprised of reads in a given family and reads outside a given family. For full models, fixed effects were the same as for the full diversity models; the same random effect was used as well. For the restricted models, we employed a zero‐inflated model by applying a zero‐inflated intercept in the Bernoulli part of the model. We used the same response variable and fixed effects as in the full model, but we were unable to include the nested random effect.

### 2.6 Differential Abundance Across Bioclimatic Zones and Land Use

To investigate differential abundance of ASVs by bioclimatic zone and land use, we used differential abundance analysis implemented via DESeq2. This allowed us to assess the differential representation of specific ASVs within families to look for variation to our overall family‐level patterns identified through our GLMMs. Specifically, we tested for differential abundance (1) between land uses for each bioclimatic zone and (2) between land uses across bioclimatic zones (temperate and tropical). For the test within bioclimatic zones, we only included biome as a fixed effect; for the test between land use across biomes, we included both land use and bioclimatic zone as fixed variables. Only ASVs with adjusted *p*‐values < 0.1 (using the Benjamini‐Hochberg correction) are reported (Table S4).

## 3 Results & Discussion

### 3.1 Identification of Novel AMF Clades

**FIGURE 1:**
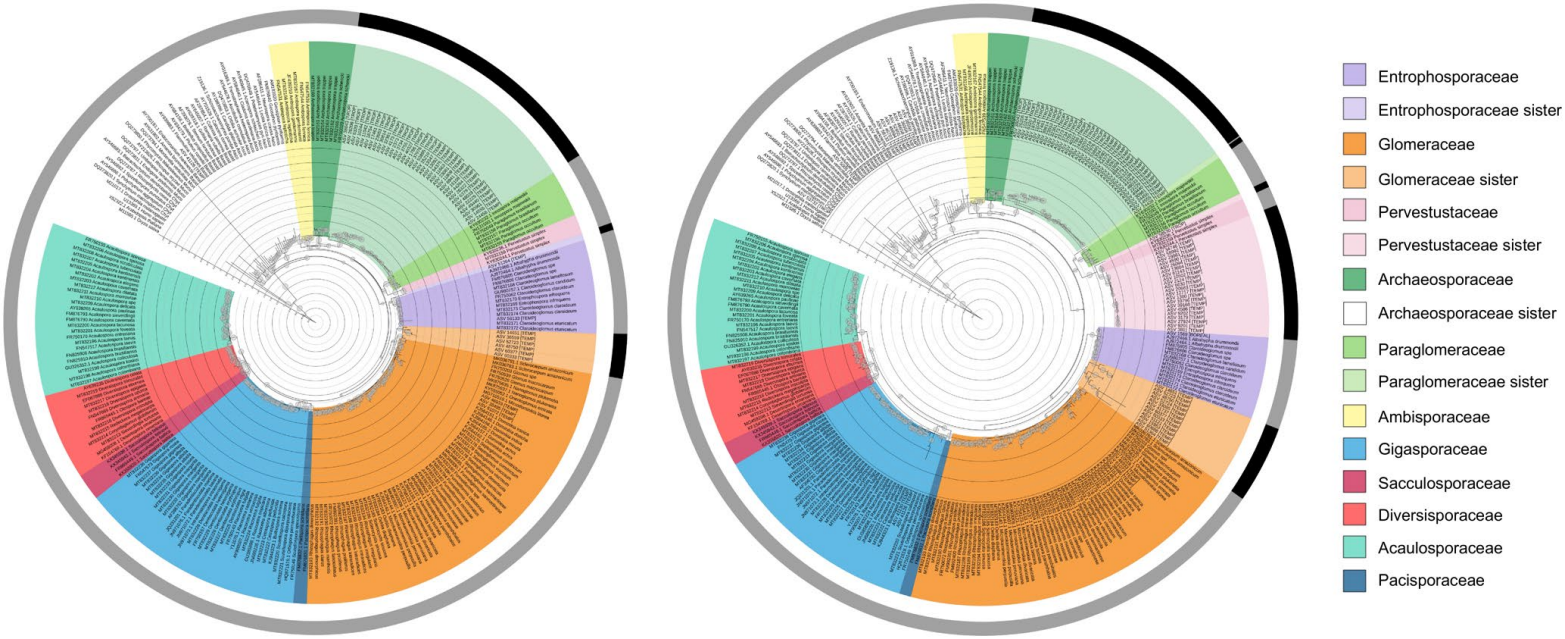
Novel arbuscular mycorrhizal fungal clades. Novel arbuscular mycorrhizal fungal (AMF) clades are identified through tree placement of taxa that could not be assigned to family using the tree placement or Basic Local Alignment Search Tool (BLAST) approaches. Placing taxa that were neither identifiable to family with the tree placement or BLAST approaches (A), representing the most conservative approach, identified three sister clades to *Entrophosporaceae*, *Glomeraceae*, and *Archaeosporaceae* families. Placing taxa that were not identifiable to family with only the tree approach (B) identified four sister clades to *Archaeosporaceae*, *Paraglomeraceae*, *Pervestustaceae*, and *Glomeraceae* families. AMF families (i.e., in the Glomeromycota clade) are shown with different color shading; outgroups have no shading; novel clades are denoted with a black edge.

Our assessment of AMF taxa across three regions within three major bioclimatic zones revealed evidence for three novel AMF clades, potentially representing previously undescribed AMF families (Figure 1 and Figure S1). In AMF ASVs placed in the Glomeromycota phylum but no family for both tree placement and BLAST approach (double unknown), we identify sister clades to the *Archaeosporaceae*, *Entrophosporaceae*, *and Glomeraceae* (Figure 1A and Figure S1A; 69%, 33%, and 26% bootstrap support, respectively). Interestingly, we find that these clades are regionally structured, with clades or subclades composed of taxa from one of our bioclimatic zones. In particular, the sister clades to *Entrophosporaceae* and *Glomeraceae* are composed of temperate taxa. The sister clade to *Archaeosporaceae* is split into two subclades, with one composed of temperate taxa and the other of tropical taxa. Recent work also found a novel clade within the *Archaeosporaceae*, from European and African samples (Kolaříková et al. 2021), suggesting that this family harbors novel diversity on several continents that has not been formally described. In the tree placing only tree unknowns (Figure 1B and Figure S1), we identify four putative clades, although one is composed of a single ASV. Specifically, we identify sister clades to *Pervestustaceae*, *Glomeraceae*, and *Paraglomeraceae*, all composed of temperate taxa, and a sister clade to *Archaeosporaceae*, again with two subclades: one composed of temperate taxa and one of tropical taxa (86%, 3%, 37%, and 60% bootstrap support). No new clades were detected from the boreal bioclimatic zone. Low recovery of AMF in this bioclimatic zone may be due to several reasons, but is not surprising given the multi‐mycorrhizal plant types coexisting in these environments. The dominance of ectomycorrhizal trees may dilute AMF density in any given amount of soil sampled. These putative AMF clades illustrate the extent of previously undiscovered AMF diversity in both temperate and tropical bioclimatic zones in which we sampled.

**FIGURE 2:**
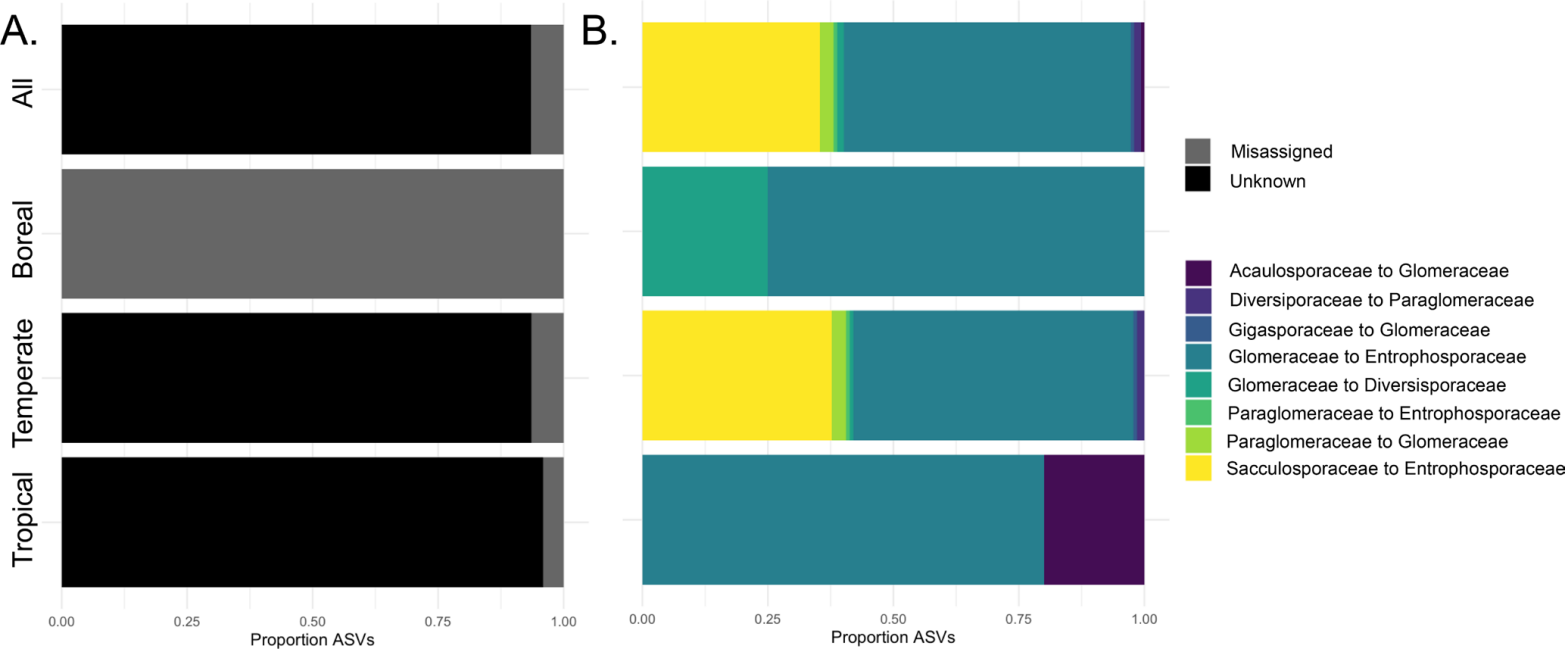
Taxa misclassified by Basic Local Alignment Search Tool. Out of 2468 ASVs, 35% of Basic Local Alignment Search Tool (BLAST) amplicon sequence variants (ASVs) were not assigned to an arbuscular mycorrhizal fungal (AMF) family (Unknown); further, 6% of BLAST ASVs were assigned to a family different than that assigned by phylogenetic placement (‘misassigned’). (A) shows the relative proportion of BLAST unknown and misassigned sequences, while (B) shows the proportion of misassigned ASVs based on the BLAST to tree AMF family, highlighting the prevalence of family level misassignment in BLAST.

Although novel clades are found regardless of which method is used for general AMF and AMF family identification, we find that the phylogenetic tree placement is preferable to BLAST, highlighting assignment errors in NCBI (Lücking et al. 2020; Bidartondo et al. 2008). The BLAST approach resulted in a high number of (1) identification of ASVs into Glomeromycota without a family assignment and (2) incorrect family assignments (Figure 2). Over one third (35%) of BLAST assigned IDs had an unknown AMF family (compared to 14.5% with the tree placement method), making family level inferences difficult with this method. In addition, when examining ASVs shared between the tree placement method and BLAST, we found misclassification, with 6% of ASVs identified as misassigned when compared to tree placement. The ASVs that are incorrectly assigned to families in BLAST occurred mostly within the *Glomeraceae* and *Entrophosporaceae* but also included taxa in the *Acaulosporaceae*, *Diversisporaceae*, and *Paraglomeraceae*. That BLAST taxonomic assignments have limited curation and erroneous naming has long been known to be a problem (Vilgalys 2003), spurring curated databases for fungi and AMF alike. Here, we confirm that the imprecision (lack of any family level assignment) and erroneous assignment (incorrect family assignment) of the BLAST approach are widespread (> 40% of data not usable at the family level) for the AMF using the large subunit (LSU) rRNA gene, making this method unreliable for detailed family level conclusions for this gene region.

These results illustrate limitations in our knowledge of AMF diversity but also highlight the power of using a phylogenetic tree method compared to the traditionally used BLAST approach for identification and characterization of AMF diversity. Moreover, these results reveal specific high priority geographic regions where additional work—at least across our sampling locations—on specific AMF families should be carried out to uncover novel taxa. We found likely novel AMF taxa within the *Entrophosporaceae*, *Glomeraceae*, *Pervestustaceae*, and *Paraglomeraceae* in temperate bioclimatic zones, at deeper depths than have been previously sampled (i.e., 1–10 cm; Figure S2). In tropical bioclimatic zones, we found likely novel AMF taxa within the *Archaeosporaceae*, despite this family being underrepresented in the tropics (see Section 3.2). Ultimately, even with our rather modest sampling, we identify parts of the AMF phylogeny that were previously unknown. Future work will need to isolate and identify these taxa morphologically for formal expansion of the AMF phylogeny, allowing for their integration into a future version of the backbone tree. In addition, long‐read sequencing (Kolaříková et al. 2021) could enable a stronger backbone tree and bootstrap support of novel clades. Broader sampling across different regions will also be needed to have a complete view of gaps in the current AMF phylogeny.

### 3.2 The Biogeography of AMF Families

**FIGURE 3:**
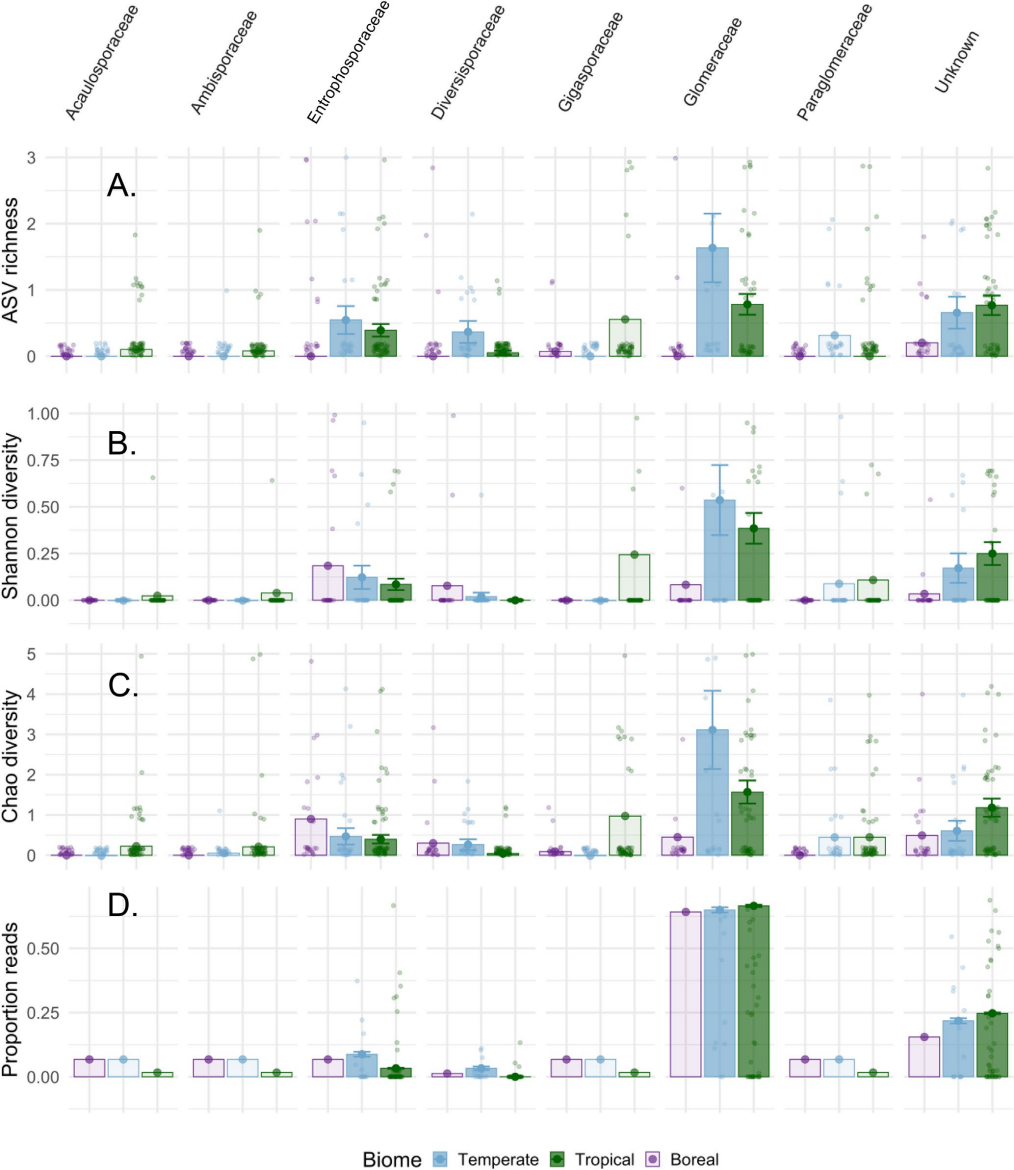
Bioclimatic zone patterns in arbuscular mycorrhizal fungal family diversity and proportion. Arbuscular mycorrhizal fungal (AMF) family (1) diversity—including ASV richness (A), Shannon diversity (B) and chao1 diversity (C)—and (2) proportion sequence reads (D) shift across bioclimatic zones. Taxa in the *Glomeraceae*, *Diversisporaceae* and *Entrophosphoraceae* are overrepresented in temperate compared to tropical samples. Proportion of reads for taxa that cannot be placed into an AMF family (unknown) is significantly higher for tropical compared to temperate systems. Estimates and standard errors are shown from restricted models (filled bars); estimates only are shown for families that only ran in the full model (transparent bars).

We find broad support for the biogeographical patterns of AMF families described previously but uncover a novel overrepresentation of AMF in the *Diversisporaceae* and *Entrophosphoraceae* in temperate compared to tropical samples (Figure 3). However, we find limited evidence for many of the AMF families, which are expected to be overrepresented in the tropics (*Acaulosporaceae*, *Gigasporaceae*, and *Ambisporaceae*), which may be due to the disproportionate number of taxa in the tropics that cannot be assigned to family. We find that there is higher diversity of *Diversisporaceae* and *Glomeraceae* in temperate compared to tropical systems (*Diversisporaceae*: ASV richness *z*‐value = −2.616, *p* = 0.009; Chao1 *z*‐value = −2.248, *p* = 0.025; BLAST *z*‐value = −2.385, *p* = 0.017; *Glomeraceae*: ASV richness *z*‐value = −2.04, *p* = 0.041, Chao1 *z*‐value = −1.931, *p* = 0.053; BLAST *z*‐value = −4.819, *p* < 0.001). In addition, the proportion of sequence reads is higher for *Entrophosphoraceae* in temperate compared to tropical systems (proportion of reads *z*‐value = −7.68, *p* < 0.001). Higher diversity within the *Glomeraceae* in temperate systems has previously been reported (Stürmer, Bever, and Morton 2018). However, overrepresentation of *Diversisporaceae* and *Entrophosporaceae* in temperate systems has not been previously shown. The discrepancy between our results and previous work may be due to methodological differences, as previous findings tested each bioclimatic zone compared to a global null model instead of using direct bioclimatic zone pairwise comparisons as we employ here. Finally, the proportion of reads for taxa that cannot be placed into an AMF family (unknown) is significantly higher for tropical compared to temperate systems (proportion of reads *z*‐value = 2.716, *p* < 0.01). Consistent with this, we find that there is a higher proportion of unknown ASVs differentially abundant in the tropical versus temperature bioclimatic zone (Table S4). This indicates that tropical systems stand out as reservoirs of unknown AMF diversity, potentially as a result of historical undersampling, and may explain why we only detect overrepresentation of AMF families within temperate systems and not in tropical systems.

Although we were unable to robustly test for differences among AMF families based on bioclimatic zones, we see a trend of higher diversity of *Acaulosporaceae*, *Ambisporaceae*, and *Gigasporaceae* in the tropics relative to other bioclimatic zones. The high representation of *Ambisporaceae* and *Gigasporaceae* agrees with previous work testing biogeography (Stürmer, Bever, and Morton 2018) and climate niches of these families (Veresoglou, Caruso, and Rillig 2013; Davison et al. 2021). The high representation of *Acaulosporaceae* in the tropics is in line with results from Stürmer, Bever, and Morton (2018), and results showing this family occurs in lower pH environments (Veresoglou, Caruso, and Rillig 2013). In contrast, other studies have shown that the niche optimum of this family occurs in low temperatures (Vasar et al. 2022; Davison et al. 2021). We also find a higher diversity of *Entrophosporaceae* and *Diversisporaceae* but a lower diversity of *Glomeraceae* and *Paraglomeraceae* in the boreal bioclimatic zone, largely confirming previous findings (Stürmer, Bever, and Morton 2018) and expanding AMF diversity relative to that previously detected in thawing permafrost soils (Schütte et al. 2019). While increasing the sample size and representation of each bioclimatic zone will be essential to assess whether these patterns are significant and consistent across bioclimatic zones, our work shows that phylogenetic placement of LSU amplicons is a promising approach for this work that avoids the limitation of eDNA approaches that target SSU or ITS (Bruns and Taylor 2016; Schütte et al. 2019).

### 3.3 Susceptibility of AMF Families to Disturbance

**FIGURE 4:**
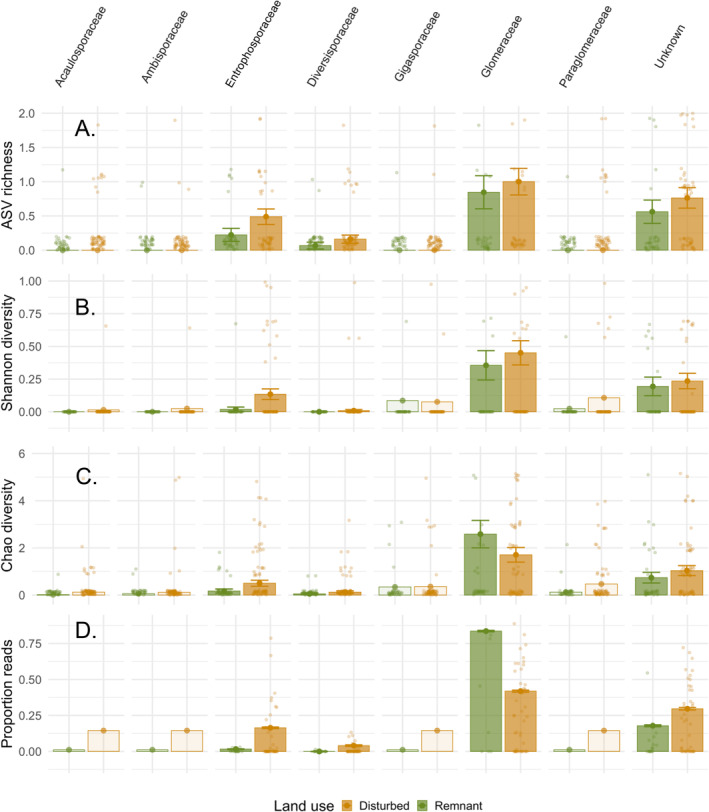
Land use patterns in arbuscular mycorrhizal fungal family diversity and proportion. Arbuscular mycorrhizal fungal (AMF) family (1) diversity—including ASV richness (A), Shannon diversity (B) and chao1 diversity (C)—and (2) proportion sequence reads (D) response to land use varies. Diversity (amplicon sequence variant richness) and proportion of reads for *Entrophosporaceae* are higher in disturbed compared to remnant site. Diversity (chao1) and proportion of reads *Glomeraceae* is lower in disturbed sites. Estimates and standard errors are shown from restricted models (filled bars); estimates only are shown for families that only ran in the full model (transparent bars).

Our results suggest that AMF diversity and proportion sequencing reads are higher in *Entrophosporaceae* in disturbed compared to remnant sites across our sampled sites spanning three climatic zones (Figure 4; Shannon *z*‐value = 1.686, *p* = 0.056; Chao1 *z*‐value = 1.957, *p* = 0.050; proportion of reads *z*‐value = 18.343, *p* < 0.001), suggesting that AMF in this family are more likely to be tolerant to disturbance compared to other families. In contrast, proportion of reads is lower in *Glomeraceae* in disturbed sites (proportion of reads *z*‐value = −39.833, *p* < 0.001), suggesting that AMF in this family may be disturbance sensitive. This perspective is supported by differential abundance analysis, as a higher proportion of taxa belonging to the *Glomeraceae* are differentially abundant in remnant versus disturbed sites (Table S4). However, we note that individual *Glomeraceae* ASVs may show the opposite pattern (Table S4), confirming the distinct biology of individual taxa within AMF families.

**FIGURE 5:**
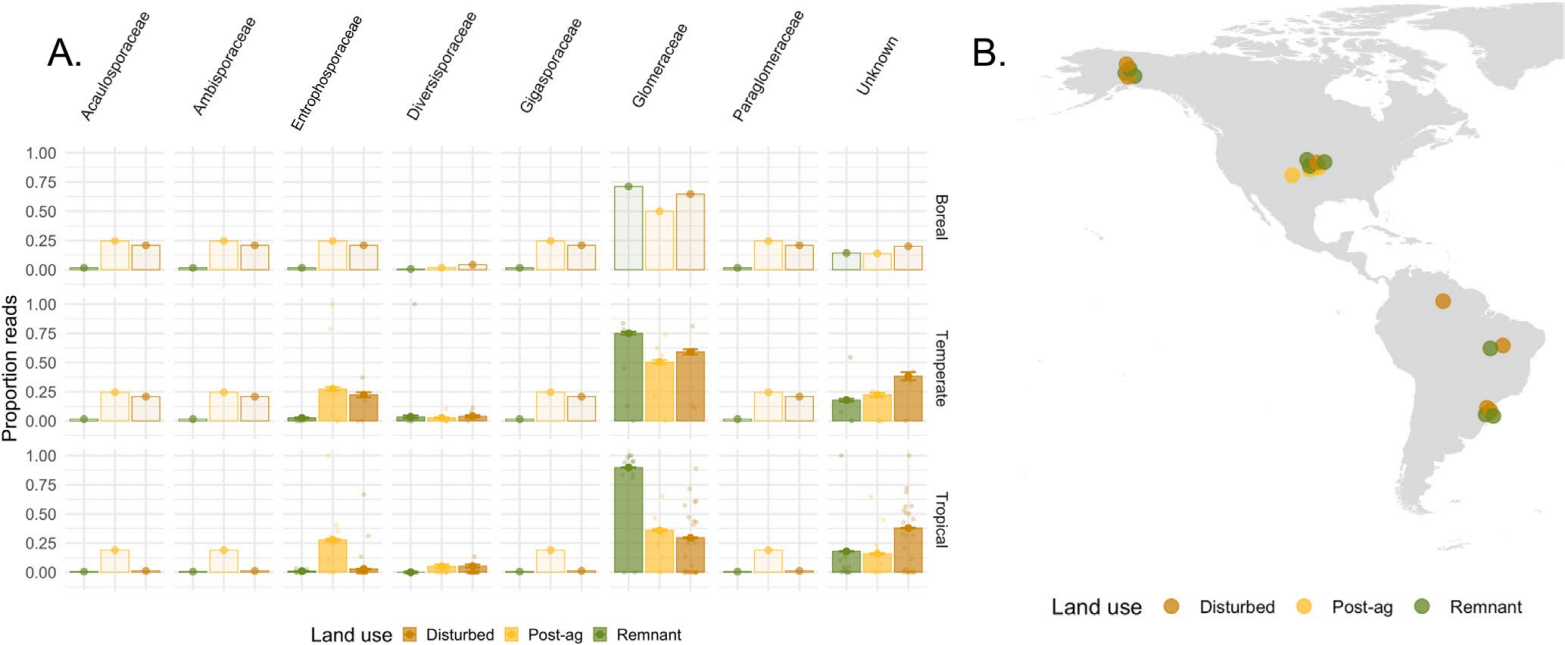
Bioclimatic zone specific land use patterns in arbuscular mycorrhizal fungal family diversity and proportion. Additional information can be gained from finer scale resolution of land use (A) to test for arbuscular mycorrhizal fungal (AMF) family level proportion of reads across bioclimatic zone (B). When testing across remnant versus disturbed sites (grouping agricultural and post‐agricultural sites), we find that *Entrophosporaceae* is overrepresented, while *Glomeraceae* is underrepresented in disturbed sites. However, testing across three land use types allows a finer scale understanding of these patterns. We find that *Entrophosporaceae* is highest in post‐agricultural systems; *Glomeraceae* is lowest in post‐agricultural systems in temperate, but in agricultural in tropical systems. Estimates and standard errors are shown from restricted models (filled bars); estimates only are shown for families that only ran in the full model (transparent bars).

When we test for differences across the three land uses, including remnant, post‐agricultural, and agricultural, we see general consistency between temperate and tropical sites for *Entrophosporaceae*, but *Glomeraceae* results are not entirely consistent (Figure 5). Across both temperate and tropical sites, *Entrophosporaceae* is overrepresented in post‐agricultural sites, with proportion of reads significantly different between all three land uses (temperate remnant vs. agricultural *z*‐value = 9.054, *p* < 0.001; agricultural vs. post‐ag *z*‐value = 1.771, *p* = 0.076; remnant vs. post‐ag *z*‐value = 10.933, *p* < 0.001; tropical remnant vs. agricultural *z*‐value = 7.088, *p* < 0.001; agricultural vs. post‐ag *z*‐value = 25.273, *p* = 0.003; remnant vs. post‐ag *z*‐value = 28.614, *p* < 0.001). *Glomeraceae* declines from remnant to agricultural to post‐agricultural sites in temperate (remnant vs. agricultural *z*‐value = −6.073, *p* < 0.001; agricultural vs. post‐ag *z*‐value = −2.979, *p* = 0.003), but from remnant to post‐agricultural to agricultural sites in tropical (remnant vs. post‐agricultural *z*‐value = −51.466, *p* < 0.001; post‐ag vs. ag *z*‐value = 5.72, *p* < 0.001). This similar pattern can be seen in diversity, but not all land uses are significantly different from one another (Table S2). These results highlight that AMF in the *Glomeraceae* are most impacted (i.e., show reduced proportion reads) in post‐agricultural sites in temperate samples, but are most impacted in agricultural sites in tropical samples. For both families, examining results across the three land use types helps refine our understanding of when they are overrepresented and whether this differs by bioclimatic zone.

Although we were unable to test for significant diversity differences between land use types, the diversity of *Paraglomeraceae* and *Diversisporaceae* was consistently higher in disturbed compared to remnant sites, suggesting these families may be disturbance‐tolerant, and even disproportionately successful in disturbed sites, agreeing with previous work (Chen et al. 2023; Cahyaningtyas and Ezawa 2023). We further observed that *Paraglomeraceae* is overrepresented, showing a higher‐proportion of sequence reads in post‐agricultural sites in both temperate and tropical systems, whereas *Diversiporaceae* is overrepresented in agricultural sites in particular. In tropical sites, *Acaulosporaceae* is overrepresented in disturbed sites as well. This result contrasts with previous work asserting that *Acaulosporaceae* is disturbance‐sensitive (Van Der Heyde et al. 2017; Oehl et al. 2003; Kajihara et al. 2022; Chagnon et al. 2013). However, this previous work may have been biased toward temperate systems, which could explain why we observe a pattern opposite that expected in our tropical sites.

Examining our results across boreal sites highlights potential future bioclimatic zone‐specific responses of AMF families to land use. *Glomeraceae*, which we identified as disturbance‐sensitive based on temperate and tropical samples, is more abundant in disturbed (agricultural) sites in our boreal samples. This suggests that from tropical and temperate systems to boreal systems, either (1) membership in this family may change to taxa with distinct disturbance sensitivities or (2) the same taxa exhibit some level of plasticity. Additionally, changes in host plant species may influence these patterns. Previous work has found support for *Glomeraceae* being both disturbance sensitive (Chen et al. 2023; House and Bever 2018) and tolerant (Van Der Heyde et al. 2017; Chagnon et al. 2013; Cahyaningtyas and Ezawa 2023). Therefore, future work is warranted to clarify the context dependency of this family and potential within family differences in response to land use. This potential variation in disturbance sensitivity may help clarify the ambiguous categorization of this family, but finer‐scale identification—to genus and species level—will continue to be informative. This highlights that focusing on the family level may not be appropriate for all AMF families. *Gigasporaceae* is another family that shows evidence of inconsistent disturbance response between bioclimatic zones. This family is more abundant in disturbed sites in temperate and tropical systems, yet more abundant in remnant sites in boreal systems. This suggests that this family could be disturbance tolerant in temperate and tropical systems but sensitive in boreal ones. The bioclimatic zone‐specific disturbance strategy of these AMF families identifies specific families to be targeted for future research.

## 4 Conclusions

This work reveals how the phylogenetic placement method can be used with LSU amplicons to identify previously undescribed components of AMF diversity. This approach can provide insights into the biogeography and land use tolerance of AMF families. Although we cannot formally erect new clades for the putative AMF clades described here, they point to potential geographic and phylogenetic regions that may harbor more Glomeromycota diversity than previously understood. Further, this work provides a methodologically independent perspective of the distribution of AMF families across the Americas, thereby contributing to our understanding of AMF. Finally, we find variation in likelihood of vulnerability to disturbance among AMF families, some of which appear to be consistent across geographic regions. This type of analysis has the potential to pinpoint at‐risk AMF families and identify those families that would benefit from reintroduction during restoration. Future work should focus on expanding representation across bioclimatic zones in the Americas and across the world and test whether family level response to disturbance varies based on bioclimatic zone. This study provides an illustrative assessment of the AMF LSU pipeline, paving the way for future work to fully describe AMF diversity, biogeography, and risk due to human land use.

## Author Contributions

**Camille S. Delavaux:** data curation (lead), formal analysis (lead), investigation (equal), project administration (lead), visualization (lead), writing – original draft (lead), writing – review and editing (equal). **Alexis Aellen:** data curation (equal), formal analysis (supporting), methodology (equal), visualization (supporting). **Sidney L. Stürmer:** conceptualization (equal), investigation (equal), methodology (equal), writing – review and editing (equal). **Silmar Primieri:** investigation (equal), writing – review and editing (equal). **Ursel M. E. Schütte:** conceptualization (equal), investigation (equal), methodology (equal), writing – review and editing (equal). **Devin M. Drown:** investigation (equal), writing – review and editing (equal). **Robert J. Ramos:** investigation (equal), methodology (equal), writing – review and editing (equal). **Thomas W. Crowther:** supervision (supporting), visualization (equal), writing – original draft (supporting), writing – review and editing (equal). **James D. Bever:** conceptualization (equal), funding acquisition (equal), investigation (equal), methodology (equal), supervision (supporting), visualization (equal), writing – original draft (equal), writing – review and editing (equal).

## Conflicts of Interest

The authors declare no conflicts of interest.

## Supporting information


Data S1.

## Data Availability

Raw sequence reads are deposited in SRA (BioProject #PRJNA1131972). All metadata can be found in the project Github repository https://github.com/c383d893/DiversityofGlomeromycota. The AMF LSU database and pipeline can be found at https://github.com/c383d893/AMF‐LSU‐Database‐and‐Pipeline2.

## References

[ece370597-bib-0001] Bates, D. , M. Maechler , B. Bolker , and S. Walker . 2015. “Fitting Linear Mixed‐Effects Models Using lme4.” Journal of Statistical Software 67: 1–48.

[ece370597-bib-0002] Bever, J. D. , J. B. Morton , J. Antonovics , and P. A. Schultz . 1996. “Host‐Dependent Sporulation and Species Diversity of Arbuscular Mycorrhizal Fungi in a Mown Grassland.” Journal of Ecology 84: 71–82.

[ece370597-bib-0003] Bidartondo, M. , T. Bruns , M. Blackwell , et al. 2008. “Preserving Accuracy in GenBank.” Science 319: 1616. 10.1126/science.319.5870.1616a 18356505

[ece370597-bib-0004] Błaszkowski, J. , M. Yamato , P. Niezgoda , et al. 2023. “A New Genus, Complexispora, With Two New Species, *C*. *multistratosa* and *C*. *mediterranea*, and *Epigeocarpum japonicum* sp. nov.” Mycological Progress 22: 34.

[ece370597-bib-0005] Brooks, M. , B. Bolker , K. Kristensen , M. Maechler , A. Magnusson , and M. Mcgillycuddy . 2023. “Package ‘glmmtmb’.” *R Packag Vers*, 1, 7.

[ece370597-bib-0006] Brownrigg, M. R. 2013. “Package ‘maps’.” *R Package*.

[ece370597-bib-0007] Brundrett, M. C. , and L. Tedersoo . 2018. “Evolutionary History of Mycorrhizal Symbioses and Global Host Plant Diversity.” New Phytologist 220: 1108–1115. 29355963 10.1111/nph.14976

[ece370597-bib-0008] Bruns, T. D. , and J. W. Taylor . 2016. “Comment on “Global Assessment of Arbuscular Mycorrhizal Fungus Diversity Reveals Very Low Endemism”.” Science 351: 826. 10.1126/science.aad4228 26912889

[ece370597-bib-0009] Cahyaningtyas, A. , and T. Ezawa . 2023. “Disturbance Tolerance of Arbuscular Mycorrhizal Fungi: Characterization of Life‐History Strategies Along a Disturbance Gradient in a Coastal Dune Ecosystem.” Plant and Soil 495: 535–549.

[ece370597-bib-0010] Chagnon, P. L. , R. L. Bradley , H. Maherali , and J. N. Klironomos . 2013. “A Trait‐Based Framework to Understand Life History of Mycorrhizal Fungi.” Trends in Plant Science 18: 484–491. 23756036 10.1016/j.tplants.2013.05.001

[ece370597-bib-0011] Chaudhary, V. , M. Lau , and N. Johnson . 2008. “Macroecology of Microbes–Biogeography of the Glomeromycota.” In Mycorrhiza, edited by A. Varma , 529–563. Berlin, Germany: Springer.

[ece370597-bib-0012] Chaudhary, V. B. , S. Nolimal , M. A. Sosa‐Hernández , C. Egan , and J. Kastens . 2020. “Trait‐Based Aerial Dispersal of Arbuscular Mycorrhizal Fungi.” New Phytologist 228: 238–252. 10.1111/nph.16667 32421866

[ece370597-bib-0013] Cheeke, T. E. , C. Zheng , L. Koziol , C. R. Gurholt , and J. D. Bever . 2019. “Sensitivity to AMF Species is Greater in Late‐Successional Than Early‐Successional Native or Nonnative Grassland Plants.” Ecology 100: e02855. 31359432 10.1002/ecy.2855 PMC6916349

[ece370597-bib-0014] Chen, K. , J. Zhang , M. A. Muneer , K. Xue , H. Niu , and B. Ji . 2023. “Plant Community and Soil Available Nutrients Drive Arbuscular Mycorrhizal Fungal Community Shifts During Alpine Meadow Degradation.” Fungal Ecology 62: 101211.

[ece370597-bib-0015] Cotton, T. A. 2018. “Arbuscular Mycorrhizal Fungal Communities and Global Change: An Uncertain Future.” FEMS Microbiology Ecology 94: fiy179. 10.1093/femsec/fiy179 30212897

[ece370597-bib-0016] Davison, J. , M. Moora , M. Öpik , et al. 2015. “Global Assessment of Arbuscular Mycorrhizal Fungus Diversity Reveals Very Low Endemism.” Science 349: 970–973. 26315436 10.1126/science.aab1161

[ece370597-bib-0017] Davison, J. , M. Moora , M. Semchenko , et al. 2021. “Temperature and pH Define the Realised Niche Space of Arbuscular Mycorrhizal Fungi.” New Phytologist 231: 763–776. 33507570 10.1111/nph.17240

[ece370597-bib-0018] Delavaux, C. S. , R. J. Ramos , S. L. Sturmer , and J. D. Bever . 2022. “Environmental Identification of Arbuscular Mycorrhizal Fungi Using the LSU rDNA Gene Region: An Expanded Database and Improved Pipeline.” Mycorrhiza 32: 145–153. 35099622 10.1007/s00572-022-01068-3 PMC8907093

[ece370597-bib-0019] Delavaux, C. S. , R. J. Ramos , S. L. Stürmer , and J. D. Bever . 2024. “An Updated LSU Database and Pipeline for Environmental DNA Identification of Arbuscular Mycorrhizal Fungi.” Mycorrhiza 34: 369–373. 38951211 10.1007/s00572-024-01159-3 PMC11283431

[ece370597-bib-0020] Delavaux, C. S. , L. M. Smith‐Ramesh , and S. E. Kuebbing . 2017. “Beyond Nutrients: A Meta‐Analysis of the Diverse Effects of Arbuscular Mycorrhizal Fungi on Plants and Soils.” Ecology 98: 2111–2119. 28500779 10.1002/ecy.1892

[ece370597-bib-0021] Delavaux, C. S. , S. L. Sturmer , M. R. Wagner , U. Schütte , J. B. Morton , and J. D. Bever . 2021. “Utility of Large Subunit for Environmental Sequencing of Arbuscular Mycorrhizal Fungi: A New Reference Database and Pipeline.” New Phytologist 229: 3048–3052. 33190292 10.1111/nph.17080

[ece370597-bib-0022] Delavaux, C. S. , P. Weigelt , W. Dawson , et al. 2021. “Mycorrhizal Types Influence Island Biogeography of Plants.” Communications Biology 4: 1–8. 34561537 10.1038/s42003-021-02649-2 PMC8463580

[ece370597-bib-0024] Fox, J. , S. Weisberg , D. Adler , et al. 2012. “Package ‘car’.” *Vienna: R Foundation for Statistical Computing*, 16, 333.

[ece370597-bib-0025] García De León, D. , M. Moora , M. Öpik , et al. 2016. “Symbiont Dynamics During Ecosystem Succession: Co‐Occurring Plant and Arbuscular Mycorrhizal Fungal Communities.” FEMS Microbiology Ecology 92: fiw097. 27162183 10.1093/femsec/fiw097

[ece370597-bib-0026] Garnier, S. , N. Ross , B. Rudis , and M. Sciaini . 2018. “Package ‘viridis’.” *Colorblind‐Friendly Color Maps for R*.

[ece370597-bib-0027] Grime, J. P. 2006. Plant Strategies, Vegetation Processes, and Ecosystem Properties. Chichester, UK: John Wiley & Sons.

[ece370597-bib-0028] Hart, M. M. , K. Aleklett , P. L. Chagnon , et al. 2015. “Navigating the Labyrinth: A Guide to Sequence‐Based, Community Ecology of Arbuscular Mycorrhizal Fungi.” New Phytologist 207: 235–247. 25737096 10.1111/nph.13340

[ece370597-bib-0029] Hart, M. M. , and R. J. Reader . 2002. “Taxonomic Basis for Variation in the Colonization Strategy of Arbuscular Mycorrhizal Fungi.” New Phytologist 153: 335–344.

[ece370597-bib-0030] Hartig, F. , and M. F. Hartig . 2017. “Package ‘dharma’.” *R package*.

[ece370597-bib-0031] House, G. L. , and J. D. Bever . 2018. “Disturbance Reduces the Differentiation of Mycorrhizal Fungal Communities in Grasslands Along a Precipitation Gradient.” Ecological Applications 28: 736–748. 29314434 10.1002/eap.1681

[ece370597-bib-0032] Johnson, N. C. , C. Angelard , I. R. Sanders , and E. T. Kiers . 2013. “Predicting Community and Ecosystem Outcomes of Mycorrhizal Responses to Global Change.” Ecology Letters 16: 140–153. 10.1111/ele.12085 23679013

[ece370597-bib-0033] Kajihara, K. T. , C. P. Egan , S. O. Swift , C. B. Wall , C. D. Muir , and N. A. Hynson . 2022. “Core Arbuscular Mycorrhizal Fungi Are Predicted by Their High Abundance–Occupancy Relationship While Host‐Specific Taxa Are Rare and Geographically Structured.” New Phytologist 234: 1464–1476. 35218016 10.1111/nph.18058

[ece370597-bib-0034] Kans, J. 2024. “Entrez Direct: e‐Utilities on the UNIX Command Line.” *Entrez Programming Utilities Help [Internet]*. National Center for Biotechnology Information (US).

[ece370597-bib-0035] Kolaříková, Z. , R. Slavíková , C. Krüger , M. Krüger , and P. Kohout . 2021. “PacBio Sequencing of Glomeromycota rDNA: A Novel Amplicon Covering all Widely Used Ribosomal Barcoding Regions and its Applicability in Taxonomy and Ecology of Arbuscular Mycorrhizal Fungi.” New Phytologist 231: 490–499. 33780549 10.1111/nph.17372

[ece370597-bib-0036] Koziol, L. , and J. D. Bever . 2016. “The Missing Link in Grassland Restoration: Arbuscular Mycorrhizal Fungi Inoculation Increases Plant Diversity and Accelerates Succession.” Journal of Applied Ecology 54: 1301–1309.

[ece370597-bib-0037] Koziol, L. , T. P. Mckenna , and J. D. Bever . 2023. “Native Microbes Amplify Native Seedling Establishment and Diversity While Inhibiting a Non‐Native Grass.” Plants 12: 1184. 36904044 10.3390/plants12051184 PMC10005557

[ece370597-bib-0038] Koziol, L. , T. P. Mckenna , T. E. Crews , and J. D. Bever . 2023. “Native Arbuscular Mycorrhizal Fungi Promote Native Grassland Diversity and Suppress Weeds 4 Years Following Inoculation.” Restoration Ecology 31: e13772.

[ece370597-bib-0039] Koziol, L. , P. A. Schultz , G. L. House , J. T. Bauer , E. L. Middleton , and J. D. Bever . 2018. “The Plant Microbiome and Native Plant Restoration: The Example of Native Mycorrhizal Fungi.” Bioscience 68: 996–1006.

[ece370597-bib-0040] Krüger, M. , C. Krüger , C. Walker , H. Stockinger , and A. Schüssle . 2012. “Phylogenetic Reference Data for Systematics and Phylotaxonomy of Arbuscular Mycorrhizal Fungi From Phylum to Species Level.” New Phytologist 193: 970–984. 22150759 10.1111/j.1469-8137.2011.03962.x

[ece370597-bib-0041] Kunzetsova, A. , P. Brockhoff , and R. Christensen . 2017. “lmerTest Package: Tests in Linear Mixed Effect Models.” Journal of Statistical Software 82: 1–26.

[ece370597-bib-0042] Lenth, R. , H. Singmann , J. Love , P. Buerkner , and M. Herve . 2019. “Package ‘emmeans’.” *R package version*, 1.

[ece370597-bib-0043] Love, M. , S. Anders , and W. Huber . 2014. “Differential Analysis of Count Data–the DESeq2 Package.” Genome Biology 15: 10–1186.

[ece370597-bib-0044] Lücking, R. , M. C. Aime , B. Robbertse , et al. 2020. “Unambiguous Identification of Fungi: Where Do We Stand and How Accurate and Precise Is Fungal DNA Barcoding?” IMA Fungus 11: 1–32. 32714773 10.1186/s43008-020-00033-z PMC7353689

[ece370597-bib-0045] Maherali, H. , and J. N. Klironomos . 2012. “Phylogenetic and Trait‐Based Assembly of Arbuscular Mycorrhizal Fungal Communities.” PLoS One 7: e36695. 22606282 10.1371/journal.pone.0036695 PMC3351463

[ece370597-bib-0046] Mangan, S. A. , E. A. Herre , and J. D. Bever . 2010. “Specificity Between Neotropical Tree Seedlings and Their Fungal Mutualists Leads to Plant–Soil Feedback.” Ecology 91: 2594–2603. 20957954 10.1890/09-0396.1

[ece370597-bib-0047] McMurdie, P. J. , and S. Holmes . 2013. “Phyloseq: An R Package for Reproducible Interactive Analysis and Graphics of Microbiome Census Data.” PLoS One 8: e61217. 23630581 10.1371/journal.pone.0061217 PMC3632530

[ece370597-bib-0048] Neuwirth, E. , and M. E. Neuwirth . 2014. “Package ‘RColorBrewer’.” *ColorBrewer Palettes*, 991.

[ece370597-bib-0049] Oehl, F. , E. Sieverding , K. Ineichen , P. Mäder , T. Boller , and A. Wiemken . 2003. “Impact of Land Use Intensity on the Species Diversity of Arbuscular Mycorrhizal Fungi in Agroecosystems of Central Europe.” Applied and Environmental Microbiology 69: 2816–2824. 12732553 10.1128/AEM.69.5.2816-2824.2003 PMC154529

[ece370597-bib-0050] Oksanen, J. , F. G. Blanchet , R. Kindt , et al. 2013. “Package ‘vegan’.” *Community Ecology Package*, *Version*, 2.

[ece370597-bib-0051] Öpik, M. , A. Vanatoa , E. Vanatoa , et al. 2010. “The Online Database MaarjAM Reveals Global and Ecosystemic Distribution Patterns in Arbuscular Mycorrhizal Fungi (Glomeromycota).” New Phytologist 188: 223–241. 20561207 10.1111/j.1469-8137.2010.03334.x

[ece370597-bib-0052] Pebesma, E. , and R. Bivand . 2023. Spatial Data Science: With Applications in R. New York: Chapman and Hall/CRC.

[ece370597-bib-0053] Roy, J. , R. Reichel , N. Brüggemann , S. Hempel , and M. C. Rillig . 2017. “Succession of Arbuscular Mycorrhizal Fungi Along a 52‐Year Agricultural Recultivation Chronosequence.” FEMS Microbiology Ecology 93: fix102. 10.1093/femsec/fix102 28922802

[ece370597-bib-0054] Schütte, U. M. , J. A. Henning , Y. Ye , et al. 2019. “Effect of Permafrost Thaw on Plant and Soil Fungal Community in a Boreal Forest: Does Fungal Community Change Mediate Plant Productivity Response?” Journal of Ecology 107: 1737–1752.

[ece370597-bib-0055] Smith, S. E. , and D. J. Read . 2008. Mycorrhizal Symbiosis. San Diego, CA: Academic Press.

[ece370597-bib-0056] Stamatakis, A. 2014. “RAxML Version 8: A Tool for Phylogenetic Analysis and Post‐Analysis of Large Phylogenies.” Bioinformatics 30: 1312–1313. 24451623 10.1093/bioinformatics/btu033 PMC3998144

[ece370597-bib-0057] Stockinger, H. , M. Krüger , and A. Schüssle . 2010. “DNA Barcoding of Arbuscular Mycorrhizal Fungi.” New Phytologist 187: 461–474. 20456046 10.1111/j.1469-8137.2010.03262.x

[ece370597-bib-0058] Stürmer, S. L. , J. D. Bever , and J. B. Morton . 2018. “Biogeography of Arbuscular Mycorrhizal Fungi (Glomeromycota): A Phylogenetic Perspective on Species Distribution Patterns.” Mycorrhiza 28: 587–603. 30187122 10.1007/s00572-018-0864-6

[ece370597-bib-0059] Team, R. C. 2019. “R: A Language and Environment for Statistical Computing.” Vienna, Austria: R Foundation for Statistical Computing.

[ece370597-bib-0060] Tedersoo, L. , M. Bahram , L. Zinger , et al. 2022. “Best Practices in Metabarcoding of Fungi: From Experimental Design to Results.” Molecular Ecology 31: 2769–2795. 35395127 10.1111/mec.16460

[ece370597-bib-0061] Tipton, A. G. , E. L. Middleton , W. G. Spollen , and C. Galen . 2019. “Anthropogenic and Soil Environmental Drivers of Arbuscular Mycorrhizal Community Composition Differ Between Grassland Ecosystems.” Botany 97: 85–99.

[ece370597-bib-0062] Tipton, A. G. , D. Nelsen , L. Koziol , et al. 2022. “Arbuscular Mycorrhizal Fungi Taxa Show Variable Patterns of Micro‐Scale Dispersal in Prairie Restorations.” Frontiers in Microbiology 13: 827293. 35935243 10.3389/fmicb.2022.827293 PMC9355535

[ece370597-bib-0063] Van Der Heyde, M. , B. Ohsowski , L. K. Abbott , and M. Hart . 2017. “Arbuscular Mycorrhizal Fungus Responses to Disturbance are Context‐Dependent.” Mycorrhiza 27: 431–440. 28120111 10.1007/s00572-016-0759-3

[ece370597-bib-0064] Varma, A. , and B. Hock . 2013. Mycorrhiza: Structure, Function, Molecular Biology and Biotechnology. Berlin, Germany: Springer Science & Business Media.

[ece370597-bib-0065] Vasar, M. , J. Davison , S.‐K. Sepp , et al. 2022. “Global Taxonomic and Phylogenetic Assembly of AM Fungi.” Mycorrhiza 32: 135–144. 35138435 10.1007/s00572-022-01072-7

[ece370597-bib-0066] Veresoglou, S. D. , T. Caruso , and M. C. Rillig . 2013. “Modelling the Environmental and Soil Factors That Shape the Niches of Two Common Arbuscular Mycorrhizal Fungal Families.” Plant and Soil 368: 507–518.

[ece370597-bib-0067] Větrovský, T. , Z. Kolaříková , C. Lepinay , et al. 2023. “GlobalAMFungi: A Global Database of Arbuscular Mycorrhizal Fungal Occurrences From High‐Throughput Sequencing Metabarcoding Studies.” New Phytologist 240: 2151–2163. 37781910 10.1111/nph.19283

[ece370597-bib-0068] Vilgalys, R. 2003. “Taxonomic Misidentification in Public DNA Databases.” New Phytologist 160: 4–5. 33873532 10.1046/j.1469-8137.2003.00894.x

[ece370597-bib-0069] Wickham, H. , M. Averick , J. Bryan , et al. 2019. “Welcome to the Tidyverse.” Journal of Open Source Software 4: 1686.

[ece370597-bib-0070] Wilke, C. O. , H. Wickham , and M. C. O. Wilke . 2019. “Package ‘cowplot’.” *Streamlined Plot Theme and Plot Annotations for ‘ggplot2’*, 1.

[ece370597-bib-0071] Zuur, A. F. , E. N. Ieno , N. J. Walker , A. A. Saveliev , and G. M. Smith . 2009. Mixed Effects Models and Extensions in Ecology With R. New York: Springer.

