# Correction to “Uncovering Diversity Within the Glomeromycota: Novel Clades, Family Distributions, and Land Use Sensitivity”

**DOI:** 10.1002/ece3.70906

**Published:** 2025-01-29

Delavaux, C. S., A. Aellen, S. L. Stürmer, et al. 2025. “Uncovering Diversity Within the Glomeromycota: Novel Clades, Family Distributions, and Land Use Sensitivity.” *Ecology and Evolution* **15**: e70597. https://doi.org/10.1002/ece3.70597.

The author name “Silmar Primier” should be changed to “Silmar Primieri.” The online version of this article has been corrected accordingly.

We apologize for this error.

