# Supplementary material for: Uncovering Diversity within the Glomeromycota: Novel Clades, Family Distributions, and Land Use Sensitivity
Source: Ecol Evol. 2025 Jan 8;15(1):e70597. doi: 10.1002/ece3.70597 (PMC11711058; doi:10.1002/ece3.70597)
Supplement: Supplementary file 1 — Data S1. [file ECE3-15-e70597-s001.docx]

**Uncovering diversity within the Glomeromycota: novel clades, family distributions and land use sensitivity**

**Author list**

*Camille S. Delavaux^1^, Alexis Aellen^1^, Sidney L. Stürmer^2^, Silmar Primier^3^, Ursel M. E. Schütte^4^, Devin M. Drown^4,5^, Robert J. Ramos^6,7,8^, Thomas W. Crowther^1^, James D. Bever^6,7^*

**Supplemental Information**

******

**Figure S1 | Novel arbuscular mycorrhizal fungal clades with bootstrap support values**

Novel arbuscular mycorrhizal fungal (AMF) clades are identified through tree placement of taxa that could not be assigned to family using the tree placement or Basic Local Alignment Search Tool (BLAST) approaches. Placing taxa that were neither identifiable to family with the tree placement or BLAST approaches, representing the most conservative approach identified three sister clades to *Entrophosporaceae*, *Glomeraceae*, and *Archaeosporaceae* families. AMF families are shown with different color shading; outgroups have no shading; clades are denoted with a black edge; bootstrap values are noted at each tree node.

**Figure S2 | Novel arbuscular mycorrhizal fungal clades annotated by depth**

Novel arbuscular mycorrhizal fungal (AMF) clades are identified through tree placement of taxa that could not be assigned to family using the tree placement or Basic Local Alignment Search Tool (BLAST) approaches. Placing taxa that were neither identifiable to family with the tree placement or BLAST approaches, representing the most conservative approach identified three sister clades to *Entrophosporaceae*, *Glomeraceae*, and *Archaeosporaceae* families. Temperate amplicon sequence variants are labelled by sampling depth. AMF families (i.e. in the are shown with different color shading; outgroups have no shading; clades are denoted with a black edge; bioclimatic zone (TEMP = temperate, TROP = tropical), land use (R = remnant, PA = post-agricultural, A = agriculturall), and sampling depth are specified in brackets at each tip.

**Table S1 | Site metadata**

This table presents metadata on the sites included in this study. The data includes geographic data (country, state, site, replicate, latitude, longitude, bioclimatic zone) as well as additional sample data. Additional sample data includes depth of sampling (cm), disturbance type (Remnant_2 and Remnant_3 describing 2 and 3 land use categories respectively; see Methods for more details), crop if site is currently or has previously been used for agriculture, and if remnant, the ecosystem.

| **Country** | **State** | **Site** | **Remnant** | **Lat** | **Long** | **Bioclimatic zone** | **Dominant plant species** | **Ecosystem** |
| --- | --- | --- | --- | --- | --- | --- | --- | --- |
| USA | Alaska | Apex_Beta | Remnant | 64.696 | -148.319 | Boreal | NA | thermokarst bog |
| USA | Alaska | Apex_Gamma | Remnant | 64.696 | -148.32 | Boreal | NA | permafrost, spruce forest |
| USA | Alaska | T.Field_Agr | Disturbed | 64.869 | -147.86 | Boreal | grass, vetch, dandelion | NA |
| USA | Alaska | T.Perm | Remnant | 64.87 | -147.86 | Boreal | NA | permafrost |
| USA | Alaska | UAF | Disturbed | 64.856 | -147.85 | Boreal | wheats, oats and barley | NA |
| USA | KS | EKS | Disturbed | 38.181 | -95.273 | Temperate | corn, soybean | NA |
| USA | KS | EKS | Post-ag | 38.181 | -95.273 | Temperate | NA | restored prairie |
| USA | KS | EKS | Remnant | 38.181 | -95.273 | Temperate | NA | tallgrass prairie |
| USA | KS | HAY | Disturbed | 38.844 | -99.317 | Temperate | wheat, sorghum, corn | NA |
| USA | KS | HAY | Post-ag | 38.844 | -99.317 | Temperate | NA | restored prairie |
| USA | KS | HAY | Remnant | 38.844 | -99.317 | Temperate | NA | tallgrass prairie |
| USA | KS | KNZ | Disturbed | 39.106 | -96.61 | Temperate | corn, soybean | NA |
| USA | KS | KNZ | Post-ag | 39.106 | -96.61 | Temperate | NA | restored prairie |
| USA | KS | KNZ | Remnant | 39.106 | -96.61 | Temperate | NA | tallgrass prairie |
| USA | KS | LVN | Post-ag | 39.261 | -95.016 | Temperate | NA | post-ag field |
| USA | KS | LVN | Remnant | 39.261 | -95.016 | Temperate | NA | tallgrass prairie |
| USA | KS | RKS | Disturbed | 39.194 | -99.176 | Temperate | corn, soybean | NA |
| USA | KS | RKS | Post-ag | 39.194 | -99.176 | Temperate | NA | restored prairie |
| USA | KS | RKS | Remnant | 39.194 | -99.176 | Temperate | NA | tallgrass prairie |
| USA | KS | SVR | Disturbed | 38.872 | -100.983 | Temperate | wheat, sorghum, corn | NA |
| USA | KS | SVR | Post-ag | 38.872 | -100.983 | Temperate | NA | post-ag field |
| USA | KS | SVR | Remnant | 38.872 | -100.983 | Temperate | NA | tallgrass prairie |
| USA | KS | TLI | Disturbed | 38.81 | -97.545 | Temperate | corn, soybean | NA |
| USA | KS | TLI | Post-ag | 38.81 | -97.545 | Temperate | NA | post-ag field |
| USA | KS | TLI | Remnant | 38.81 | -97.545 | Temperate | NA | tallgrass prairie |
| USA | KS | TRB | Disturbed | 38.47 | -101.783 | Temperate | wheat, sorghum | NA |
| USA | KS | TRB | Post-ag | 38.47 | -101.783 | Temperate | NA | restored prairie |
| USA | KS | TRB | Remnant | 38.47 | -101.783 | Temperate | NA | tallgrass prairie |
| Brazil | Roraima | RR-Adv4 | Disturbed | 2.25 | -60.665 | Tropical | green manure crops (Canavalia ensiformis, Cajanus cajan, Crotalaria juncea, C. spectabilis, Stylosanthes sp., and Mucuna pruriens) | NA |
| Brazil | Roraima | RR-Adv5 | Disturbed | 2.25 | -60.665 | Tropical | green manure crops (Canavalia ensiformis, Cajanus cajan, Crotalaria juncea, C. spectabilis, Stylosanthes sp., and Mucuna pruriens) | NA |
| Brazil | Roraima | RR-Adv6 | Disturbed | 2.25 | -60.665 | Tropical | green manure crops (Canavalia ensiformis, Cajanus cajan, Crotalaria juncea, C. spectabilis, Stylosanthes sp., and Mucuna pruriens) | NA |
| Brazil | Roraima | RR-Adv7 | Disturbed | 2.25 | -60.665 | Tropical | green manure crops (Canavalia ensiformis, Cajanus cajan, Crotalaria juncea, C. spectabilis, Stylosanthes sp., and Mucuna pruriens) | NA |
| Brazil | Roraima | RR-FL5 | Disturbed | 2.25 | -60.665 | Tropical | acacia and eucalyptus plantation | NA |
| Brazil | Roraima | RR-FL6 | Disturbed | 2.25 | -60.665 | Tropical | acacia and eucalyptus plantation | NA |
| Brazil | Roraima | RR-FL7 | Disturbed | 2.25 | -60.665 | Tropical | acacia and eucalyptus plantation | NA |
| Brazil | Roraima | RR-FL8 | Disturbed | 2.25 | -60.665 | Tropical | acacia and eucalyptus plantation | NA |
| Brazil | Roraima | RR-FL9 | Disturbed | 2.25 | -60.665 | Tropical | acacia and eucalyptus plantation | NA |
| Brazil | Roraima | RR-Nat1 | Remnant | 2.25 | -60.665 | Tropical | NA | native grasslands |
| Brazil | Roraima | RR-Nat2 | Remnant | 2.25 | -60.665 | Tropical | NA | native grasslands |
| Brazil | Roraima | RR-Nat3 | Remnant | 2.25 | -60.665 | Tropical | NA | native grasslands |
| Brazil | Roraima | RR-Nat4 | Remnant | 2.25 | -60.665 | Tropical | NA | native grasslands |
| Brazil | Roraima | RR-Nat5 | Remnant | 2.25 | -60.665 | Tropical | NA | native grasslands |
| Brazil | Roraima | RR-Plt2 | Disturbed | 2.25 | -60.665 | Tropical | crop rotation with corn, soybean and rice | NA |
| Brazil | Roraima | RR-Plt3 | Disturbed | 2.25 | -60.665 | Tropical | crop rotation with corn, soybean and rice | NA |
| Brazil | Roraima | RR-Plt4 | Disturbed | 2.25 | -60.665 | Tropical | crop rotation with corn, soybean and rice | NA |
| Brazil | Roraima | RR-Plt5 | Disturbed | 2.25 | -60.665 | Tropical | crop rotation with corn, soybean and rice | NA |
| Brazil | Roraima | RR-Plt6 | Disturbed | 2.25 | -60.665 | Tropical | crop rotation with corn, soybean and rice | NA |
| Brazil | Roraima | RR-Ps3 | Post-ag | 2.25 | -60.665 | Tropical | fallow with grass Brachiaria ruziziensis | NA |
| Brazil | Santa_Catarina | LgA | Disturbed | -27.761 | -50.082 | Tropical | corn, soybean | NA |
| Brazil | Santa_Catarina | LgB | Post-ag | -27.762 | -50.081 | Tropical | fallow | NA |
| Brazil | Santa_Catarina | LgC | Remnant | -27.761 | -50.076 | Tropical | NA | native grasslands |
| Brazil | Santa_Catarina | LgF | Disturbed | -27.752 | -50.079 | Tropical | pinus plantation | NA |
| Brazil | Santa_Catarina | LgP | Remnant | -27.761 | -50.078 | Tropical | Araucaria angustifolia | NA |
| Brazil | Tocantins | Ag | Disturbed | -10.402 | -48.36 | Tropical | soybean | NA |
| Brazil | Tocantins | Na | Remnant | -10.144 | -48.317 | Tropical | NA | native Cerado area |

**Table S2 | Results from models testing for drivers of arbuscular mycorrhizal family level diversity**

Contrasts from generalized linear models predicting one of three diversity metrics for tree based data using two land use categories (A), A, but using three land use categories (B), and using Basic Local Alignment Search Tool (BLAST) assignments (C). For each model, manual contrasts were created to test for differences in diversity metrics (1) based on bioclimatic zone and (2) land use, each across all arbuscular mycorrhizal fungal (AMF) families and for each AMF family individually.

| **A. TREE** | | | | | |
| --- | --- | --- | --- | --- | --- |
| ASV richness | | | | | |
| Bioclimatic zone contrasts | | | | | |
| observed ~ AMF_family*Biome + reads_sample + (1\|Biome:State:Site:Replicate) | | | | | |
| contrast | estimate | SE | df | z ratio | p-value |
| Temp v Trop | -2.859 | 1.385 | Inf | -2.064 | **0.039** |
| Temp v Trop Cla | -0.334 | 0.450 | Inf | -0.741 | 0.459 |
| Temp v Trop Div | -1.946 | 0.744 | Inf | -2.616 | **0.009** |
| Temp v Trop Glo | -0.736 | 0.361 | Inf | -2.040 | **0.041** |
| Temp v Trop Unk | 0.157 | 0.409 | Inf | 0.385 | 0.700 |
| Land use contrasts | | | | | |
| observed ~ AMF_family*Remnant_2 + reads_sample + (1\|Biome:State:Site:Replicate) | | | | | |
| contrast | estimate | SE | df | z ratio | p-value |
| Remnant v Disturbed | 2.134 | 1.116 | Inf | 1.911 | **0.056** |
| Remnant v Disturbed Cla | 0.785 | 0.465 | Inf | 1.686 | 0.092 |
| Remnant v Disturbed Div | 0.874 | 0.806 | Inf | 1.084 | 0.278 |
| Remnant v Disturbed Glo | 0.169 | 0.301 | Inf | 0.561 | 0.575 |
| Remnant v Disturbed Unk | 0.306 | 0.331 | Inf | 0.926 | 0.354 |
| Shannon diversity | | | | | |
| Bioclimatic zone contrasts | | | | | |
| shannon ~ AMF_family*Biome + (1\|Biome:State:Site:Replicate) | | | | | |
| contrast | estimate | SE | df | z ratio | p-value |
| Temp v Trop | -20.104 | 7656.103 | Inf | -0.003 | 0.998 |
| Temp v Trop Cla | -0.367 | 0.612 | Inf | -0.600 | 0.549 |
| Temp v Trop Div | -19.779 | 7656.103 | Inf | -0.003 | 0.998 |
| Temp v Trop Glo | -0.331 | 0.398 | Inf | -0.830 | 0.406 |
| Temp v Trop Unk | 0.373 | 0.506 | Inf | 0.736 | 0.462 |
| Land use contrasts | | | | | |
| shannon ~ AMF_family*Remnant_2 + (1\|Biome:State:Site:Replicate) | | | | | |
| contrast | estimate | SE | df | z ratio | p-value |
| Remnant v Disturbed | 20.562 | 7519.695 | Inf | 0.003 | 0.998 |
| Remnant v Disturbed Cla | 1.990 | 0.994 | Inf | 2.001 | **0.045** |
| Remnant v Disturbed Div | 18.140 | 7519.694 | Inf | 0.002 | 0.998 |
| Remnant v Disturbed Glo | 0.238 | 0.344 | Inf | 0.692 | 0.489 |
| Remnant v Disturbed Unk | 0.193 | 0.407 | Inf | 0.476 | 0.634 |
| Chao1 diversity | | | | | |
| Bioclimatic zone contrasts | | | | | |
| chao1 ~ AMF_family*Biome + (1\|Biome:State:Site:Replicate) | | | | | |
| contrast | estimate | SE | df | z ratio | p-value |
| Temp v Trop | -2.039 | 1.494 | Inf | -1.365 | 0.172 |
| Temp v Trop Cla | -0.164 | 0.508 | Inf | -0.323 | 0.746 |
| Temp v Trop Div | -1.855 | 0.825 | Inf | -2.248 | **0.025** |
| Temp v Trop Glo | -0.685 | 0.355 | Inf | -1.931 | **0.053** |
| Temp v Trop Unk | 0.665 | 0.446 | Inf | 1.492 | 0.136 |
| Land use contrasts | | | | | |
| chao1 ~ AMF_family*Remnant_2 + (1\|Biome:State:Site:Replicate) | | | | | |
| contrast | estimate | SE | df | z ratio | p-value |
| Remnant v Disturbed | 1.867 | 1.208 | Inf | 1.546 | 0.122 |
| Remnant v Disturbed Cla | 1.069 | 0.546 | Inf | 1.957 | **0.050** |
| Remnant v Disturbed Div | 0.874 | 0.902 | Inf | 0.969 | 0.333 |
| Remnant v Disturbed Glo | -0.415 | 0.249 | Inf | -1.668 | 0.095 |
| Remnant v Disturbed Unk | 0.339 | 0.336 | Inf | 1.007 | 0.314 |
| **B. TREE - 3 land use category** | | | | | |
| ASV richness | | | | | |
| Land use contrasts | | | | | |
| observed ~ AMF_family*Remnant + reads_sample + (1\|Biome:State:Site:Replicate) | | | | | |
| contrast | estimate | SE | df | z ratio | p-value |
| Remnant v Disturbed | 1.643 | 1.218 | Inf | 1.349 | 0.177 |
| Remnant v Post-ag | 3.033 | 1.253 | Inf | 2.421 | **0.016** |
| Post-ag v Disturbed | -1.389 | 1.073 | Inf | -1.295 | 0.195 |
| Remnant v Disturbed Cla | 0.578 | 0.506 | Inf | 1.144 | 0.253 |
| Remnant v Disturbed Div | 0.759 | 0.861 | Inf | 0.881 | 0.378 |
| Remnant v Disturbed Glo | -0.034 | 0.312 | Inf | -0.109 | 0.913 |
| Remnant v Disturbed Unk | 0.340 | 0.349 | Inf | 0.976 | 0.329 |
| Remnant v Post-ag Cla | 1.071 | 0.500 | Inf | 2.142 | **0.032** |
| Remnant v Post-ag Div | 1.092 | 0.894 | Inf | 1.221 | 0.222 |
| Remnant v Post-ag Glo | 0.618 | 0.342 | Inf | 1.807 | 0.071 |
| Remnant v Post-ag Unk | 0.252 | 0.388 | Inf | 0.648 | 0.517 |
| Post-ag v Disturbed Cla | -0.493 | 0.410 | Inf | -1.200 | 0.230 |
| Post-ag v Disturbed Div | -0.333 | 0.705 | Inf | -0.473 | 0.637 |
| Post-ag v Disturbed Glo | -0.652 | 0.305 | Inf | -2.140 | **0.032** |
| Post-ag v Disturbed Unk | 0.089 | 0.354 | Inf | 0.251 | 0.802 |
| Shannon diversity | | | | | |
| Land use contrasts | | | | | |
| shannon ~ AMF_family*Remnant + (1\|Biome:State:Site:Replicate) | | | | | |
| contrast | estimate | SE | df | z ratio | p-value |
| Remnant v Disturbed | 4.847 | ######## | Inf | 0.000 | 1.000 |
| Remnant v Post-ag | 26.082 | ######## | Inf | 0.001 | 1.000 |
| Post-ag v Disturbed | -21.235 | 8800.310 | Inf | -0.002 | 0.998 |
| Remnant v Disturbed Cla | 1.535 | 1.050 | Inf | 1.465 | 0.143 |
| Remnant v Disturbed Div | 3.398 | ######## | Inf | 0.000 | 1.000 |
| Remnant v Disturbed Glo | -0.343 | 0.340 | Inf | -1.007 | 0.314 |
| Remnant v Disturbed Unk | 0.257 | 0.420 | Inf | 0.616 | 0.538 |
| Remnant v Post-ag Cla | 2.533 | 1.030 | Inf | 2.464 | **0.014** |
| Remnant v Post-ag Div | 23.148 | ######## | Inf | 0.000 | 1.000 |
| Remnant v Post-ag Glo | 0.289 | 0.350 | Inf | 0.833 | 0.405 |
| Remnant v Post-ag Unk | 0.113 | 0.500 | Inf | 0.227 | 0.820 |
| Post-ag v Disturbed Cla | -0.998 | 0.570 | Inf | -1.751 | 0.080 |
| Post-ag v Disturbed Div | -19.749 | 8800.310 | Inf | -0.002 | 0.998 |
| Post-ag v Disturbed Glo | -0.632 | 0.340 | Inf | -1.837 | 0.066 |
| Post-ag v Disturbed Unk | 0.144 | 0.460 | Inf | 0.313 | 0.754 |
| Chao1 diversity | | | | | |
| Land use contrasts | | | | | |
| chao1 ~ AMF_family*Remnant + (1\|Biome:State:Site:Replicate) | | | | | |
| contrast | estimate | SE | df | z ratio | p-value |
| Remnant v Disturbed | 1.188 | 1.307 | Inf | 0.909 | 0.364 |
| Remnant v Post-ag | 2.506 | 1.363 | Inf | 1.839 | 0.066 |
| Post-ag v Disturbed | -1.319 | 1.172 | Inf | -1.125 | 0.261 |
| Remnant v Disturbed Cla | 0.636 | 0.597 | Inf | 1.066 | 0.287 |
| Remnant v Disturbed Div | 0.739 | 0.961 | Inf | 0.770 | 0.442 |
| Remnant v Disturbed Glo | -0.630 | 0.281 | Inf | -2.241 | **0.025** |
| Remnant v Disturbed Unk | 0.442 | 0.355 | Inf | 1.247 | 0.213 |
| Remnant v Post-ag Cla | 1.541 | 0.581 | Inf | 2.651 | **0.008** |
| Remnant v Post-ag Div | 1.049 | 1.012 | Inf | 1.036 | 0.300 |
| Remnant v Post-ag Glo | -0.148 | 0.293 | Inf | -0.506 | 0.613 |
| Remnant v Post-ag Unk | 0.065 | 0.423 | Inf | 0.153 | 0.878 |
| Post-ag v Disturbed Cla | -0.905 | 0.464 | Inf | -1.953 | 0.051 |
| Post-ag v Disturbed Div | -0.309 | 0.812 | Inf | -0.381 | 0.704 |
| Post-ag v Disturbed Glo | -0.481 | 0.297 | Inf | -1.619 | 0.105 |
| Post-ag v Disturbed Unk | 0.377 | 0.385 | Inf | 0.981 | 0.327 |
| **C. BLAST** | | | | | |
| ASV richness | | | | | |
| Bioclimatic zone contrasts | | | | | |
| observed ~ AMF_family*Biome + reads_sample + (1\|Biome:State:Site:Replicate) | | | | | |
| contrast | estimate | SE | df | z ratio | p-value |
| Temp v Trop | -7.307 | 1.797 | Inf | -4.066 | **<.0001** |
| Temp v Trop Arc | -1.671 | 0.512 | Inf | -3.264 | **0.001** |
| Temp v Trop Cla | -1.675 | 0.773 | Inf | -2.168 | **0.030** |
| Temp v Trop Div | -1.807 | 0.757 | Inf | -2.385 | **0.017** |
| Temp v Trop Glo | -1.617 | 0.336 | Inf | -4.819 | **<.0001** |
| Temp v Trop Unk | -0.536 | 0.321 | Inf | -1.672 | 0.095 |
| Land use contrasts | | | | | |
| observed ~ AMF_family*Remnant_2 + reads_sample + (1\|Biome:State:Site:Replicate) | | | | | |
| contrast | estimate | SE | df | z ratio | p-value |
| Remnant v Disturbed | -3.146 | 1.602 | Inf | -1.963 | **0.050** |
| Remnant v Disturbed Arc | 2.553 | 1.037 | Inf | 2.462 | **0.014** |
| Remnant v Disturbed Cla | 0.605 | 0.825 | Inf | 0.734 | 0.463 |
| Remnant v Disturbed Div | 0.202 | 0.716 | Inf | 0.282 | 0.778 |
| Remnant v Disturbed Glo | -0.058 | 0.224 | Inf | -0.259 | 0.796 |
| Remnant v Disturbed Unk | -0.157 | 0.220 | Inf | -0.714 | 0.475 |
| Shannon diversity | | | | | |
| Bioclimatic zone contrasts | | | | | |
| shannon ~ AMF_family*Biome + (1\|Biome:State:Site:Replicate) | | | | | |
| contrast | estimate | SE | df | z ratio | p-value |
| Temp v Trop | -26.213 | 6848.655 | Inf | -0.004 | 0.997 |
| Temp v Trop Arc | -1.495 | 0.701 | Inf | -2.133 | **0.033** |
| Temp v Trop Cla | -1.411 | 1.037 | Inf | -1.361 | 0.174 |
| Temp v Trop Div | -22.082 | 6848.655 | Inf | -0.003 | 0.997 |
| Temp v Trop Glo | -1.086 | 0.314 | Inf | -3.460 | **0.001** |
| Temp v Trop Unk | -0.140 | 0.272 | Inf | -0.515 | 0.606 |
| Land use contrasts | | | | | |
| shannon ~ AMF_family*Remnant_2 | | | | | |
| contrast | estimate | SE | df | z ratio | p-value |
| Remnant v Disturbed | 2.836 | ######## | Inf | 0.000 | 1.000 |
| Remnant v Disturbed Arc | 22.601 | 6034.204 | Inf | 0.004 | 0.997 |
| Remnant v Disturbed Cla | -0.185 | 1.056 | Inf | -0.175 | 0.861 |
| Remnant v Disturbed Div | -24.864 | ######## | Inf | -0.001 | 0.999 |
| Remnant v Disturbed Glo | -0.343 | 0.283 | Inf | -1.213 | 0.225 |
| Remnant v Disturbed Unk | -0.046 | 0.222 | Inf | -0.205 | 0.838 |
| Chao1 diversity | | | | | |
| Bioclimatic zone contrasts | | | | | |
| chao1 ~ AMF_family*Biome + (1\|Biome:State:Site:Replicate) | | | | | |
| contrast | estimate | SE | df | z ratio | p-value |
| Temp v Trop | -7.982 | 1.966 | Inf | -4.060 | **<.0001** |
| Temp v Trop Arc | -1.663 | 0.631 | Inf | -2.635 | **0.008** |
| Temp v Trop Cla | -1.724 | 0.888 | Inf | -1.941 | **0.052** |
| Temp v Trop Div | -1.827 | 0.878 | Inf | -2.081 | **0.038** |
| Temp v Trop Glo | -2.281 | 0.353 | Inf | -6.463 | **<.0001** |
| Temp v Trop Unk | -0.487 | 0.335 | Inf | -1.454 | 0.146 |
| Land use contrasts | | | | | |
| chao1 ~ AMF_family*Remnant_2 + (1\|Biome:State:Site:Replicate) | | | | | |
| contrast | estimate | SE | df | z ratio | p-value |
| Remnant v Disturbed | -3.861 | 1.786 | Inf | -2.161 | **0.031** |
| Remnant v Disturbed Arc | 2.759 | 1.129 | Inf | 2.444 | **0.015** |
| Remnant v Disturbed Cla | 0.515 | 0.932 | Inf | 0.553 | 0.580 |
| Remnant v Disturbed Div | 0.216 | 0.875 | Inf | 0.247 | 0.805 |
| Remnant v Disturbed Glo | 0.483 | 0.249 | Inf | 1.942 | **0.052** |
| Remnant v Disturbed Unk | -0.112 | 0.222 | Inf | -0.504 | 0.614 |

**Table S3 | Results from models testing for drivers of arbuscular mycorrhizal family level sequence read proportions**

Contrasts from generalized linear models predicting one of three proportion sequence reads for tree based data using two land use categories (A), A, but using three land use categories (B), and A, using Basic Local Alignment Search Tool (BLAST) assignments. For each model, manual contrasts were created to test for differences in proportion sequence reads (1) based on bioclimatic zone and (2) land use, each across all arbuscular mycorrhizal fungal (AMF) families and for each AMF family individually.

| **A. TREE** | | | | | |
| --- | --- | --- | --- | --- | --- |
| cbind(reads_sample,rest) ~ Biome*AMF_family*Remnant_2 | | | | | |
| contrast | estimate | SE | df | z ratio | p-value |
| Temp v Trop | -19.947 | 817.354 | Inf | -0.024 | 0.981 |
| Remnant v Disturbed | 21.539 | 817.354 | Inf | 0.026 | 0.979 |
| Temp v Trop Cla | -2.065 | 0.269 | Inf | -7.680 | **<.0001** |
| Temp v Trop Div | -18.352 | 817.354 | Inf | -0.022 | 0.982 |
| Temp v Trop Glo | 0.142 | 0.098 | Inf | 1.442 | 0.149 |
| Temp v Trop Unk | 0.328 | 0.121 | Inf | 2.716 | **0.007** |
| Remnant v Disturbed Cla | 4.932 | 0.269 | Inf | 18.343 | **<.0001** |
| Remnant v Disturbed Div | 19.198 | 817.354 | Inf | 0.023 | 0.981 |
| Remnant v Disturbed Glo | -3.915 | 0.098 | Inf | -39.833 | **<.0001** |
| Remnant v Disturbed Unk | 1.324 | 0.121 | Inf | 10.955 | **<.0001** |
| Remnant v Disturbed Cla Temp | 2.584 | 0.240 | Inf | 10.775 | **<.0001** |
| Remnant v Disturbed Div Temp | -0.118 | 0.421 | Inf | -0.281 | 0.778 |
| Remnant v Disturbed Glo Temp | -0.966 | 0.090 | Inf | -10.727 | **<.0001** |
| Remnant v Disturbed Unk Temp | 0.503 | 0.117 | Inf | 4.309 | **<.0001** |
| Remnant v Disturbed Cla Trop | 2.348 | 0.122 | Inf | 19.315 | **<.0001** |
| Remnant v Disturbed Div Trop | 19.316 | 817.353 | Inf | 0.024 | 0.981 |
| Remnant v Disturbed Glo Trop | -2.949 | 0.040 | Inf | -74.728 | **<.0001** |
| Remnant v Disturbed Unk Trop | 0.821 | 0.031 | Inf | 26.197 | **<.0001** |
| **B. TREE - 3 land use categories** | | | | | |
| cbind(reads_sample,rest) ~ Biome*AMF_family*Remnant | | | | | |
| contrast | estimate | SE | df | z ratio | p-value |
| Temp v Trop | -22.814 | 1058.247 | Inf | -0.022 | 0.983 |
| Remnant v Disturbed | 21.730 | 1058.247 | Inf | 0.021 | 0.984 |
| Remnant v Post-Ag | 22.079 | 1058.247 | Inf | 0.021 | 0.983 |
| Disturbed v Post-Ag | 0.349 | 0.642 | Inf | 0.543 | 0.587 |
| Temp v Trop Cla | -3.183 | 0.315 | Inf | -10.099 | **<.0001** |
| Temp v Trop Div | -18.411 | 1058.247 | Inf | -0.017 | 0.986 |
| Temp v Trop Glo | -0.778 | 0.150 | Inf | -5.183 | **<.0001** |
| Temp v Trop Unk | -0.442 | 0.208 | Inf | -2.130 | **0.033** |
| Remnant v Disturbed Cla | 3.414 | 0.301 | Inf | 11.343 | **<.0001** |
| Remnant v Disturbed Div | 19.997 | 1058.247 | Inf | 0.019 | 0.985 |
| Remnant v Disturbed Glo | -3.764 | 0.128 | Inf | -29.533 | **<.0001** |
| Remnant v Disturbed Unk | 2.084 | 0.173 | Inf | 12.057 | **<.0001** |
| Remnant v Post-Ag Cla | 6.250 | 0.274 | Inf | 22.831 | **<.0001** |
| Remnant v Post-Ag Div | 19.518 | 1058.247 | Inf | 0.018 | 0.985 |
| Remnant v Post-Ag Glo | -3.822 | 0.111 | Inf | -34.325 | **<.0001** |
| Remnant v Post-Ag Unk | 0.133 | 0.144 | Inf | 0.922 | 0.357 |
| Disturbed v Post-Ag Cla | 2.836 | 0.182 | Inf | 15.567 | **<.0001** |
| Disturbed v Post-Ag Div | -0.479 | 0.572 | Inf | -0.837 | 0.403 |
| Disturbed v Post-Ag Glo | -0.058 | 0.128 | Inf | -0.449 | 0.653 |
| Disturbed v Post-Ag Unk | -1.951 | 0.189 | Inf | -10.326 | **<.0001** |
| Remnant v Disturbed Cla Temp | 2.392 | 0.264 | Inf | 9.054 | **<.0001** |
| Remnant v Disturbed Div Temp | 0.139 | 0.463 | Inf | 0.301 | 0.764 |
| Remnant v Disturbed Glo Temp | -0.730 | 0.120 | Inf | -6.073 | **<.0001** |
| Remnant v Disturbed Unk Temp | 1.054 | 0.170 | Inf | 6.210 | **<.0001** |
| Remnant v Post-Ag Cla Temp | 2.660 | 0.243 | Inf | 10.933 | **<.0001** |
| Remnant v Post-Ag Div Temp | -0.311 | 0.458 | Inf | -0.680 | 0.497 |
| Remnant v Post-Ag Glo Temp | -1.080 | 0.098 | Inf | -11.046 | **<.0001** |
| Remnant v Post-Ag Unk Temp | 0.283 | 0.130 | Inf | 2.173 | **0.030** |
| Disturbed v Post-Ag Cla Temp | 0.268 | 0.151 | Inf | 1.771 | 0.077 |
| Disturbed v Post-Ag Div Temp | -0.450 | 0.372 | Inf | -1.209 | 0.227 |
| Disturbed v Post-Ag Glo Temp | -0.350 | 0.117 | Inf | -2.979 | **0.003** |
| Disturbed v Post-Ag Unk Temp | -0.771 | 0.179 | Inf | -4.307 | **<.0001** |
| Remnant v Disturbed Cla Trop | 1.022 | 0.144 | Inf | 7.088 | **<.0001** |
| Remnant v Disturbed Div Trop | 19.857 | 1058.247 | Inf | 0.019 | 0.985 |
| Remnant v Disturbed Glo Trop | -3.034 | 0.042 | Inf | -71.606 | **<.0001** |
| Remnant v Disturbed Unk Trop | 1.030 | 0.033 | Inf | 31.558 | **<.0001** |
| Remnant v Post-Ag Cla Trop | 3.590 | 0.126 | Inf | 28.614 | **<.0001** |
| Remnant v Post-Ag Div Trop | 19.829 | 1058.247 | Inf | 0.019 | 0.985 |
| Remnant v Post-Ag Glo Trop | -2.742 | 0.053 | Inf | -51.466 | **<.0001** |
| Remnant v Post-Ag Unk Trop | -0.150 | 0.061 | Inf | -2.458 | **0.014** |
| Disturbed v Post-Ag Cla Trop | 2.568 | 0.102 | Inf | 25.273 | **<.0001** |
| Disturbed v Post-Ag Div Trop | -0.029 | 0.435 | Inf | -0.066 | 0.948 |
| Disturbed v Post-Ag Glo Trop | 0.292 | 0.051 | Inf | 5.720 | **<.0001** |
| Disturbed v Post-Ag Unk Trop | -1.180 | 0.061 | Inf | -19.480 | **<.0001** |
| **C. BLAST** | | | | | |
| cbind(reads_sample,rest) ~ Biome*AMF_family*Remnant_2 + (1\|Biome:State:Site:Replicate) | | | | | |
| contrast | estimate | SE | df | z ratio | p-value |
| Temp v Trop | -56.002 | 60009.190 | Inf | -0.001 | 0.999 |
| Remnant v Disturbed | 70.905 | 60009.190 | Inf | 0.001 | 0.999 |
| Temp v Trop Arc | -12.420 | 298.860 | Inf | -0.042 | 0.967 |
| Temp v Trop Cla | -26.425 | 56905.150 | Inf | 0.000 | 1.000 |
| Temp v Trop Div | -22.546 | 19047.750 | Inf | -0.001 | 0.999 |
| Temp v Trop Glo | 1.689 | 1.940 | Inf | 0.869 | 0.385 |
| Temp v Trop Unk | 3.700 | 1.940 | Inf | 1.903 | 0.057 |
| Remnant v Disturbed Arc | 22.673 | 298.860 | Inf | 0.076 | 0.940 |
| Remnant v Disturbed Cla | 25.825 | 56905.150 | Inf | 0.000 | 1.000 |
| Remnant v Disturbed Div | 23.902 | 19047.750 | Inf | 0.001 | 0.999 |
| Remnant v Disturbed Glo | -0.167 | 0.790 | Inf | -0.210 | 0.834 |
| Remnant v Disturbed Unk | -1.328 | 0.790 | Inf | -1.673 | 0.094 |
| Remnant v Disturbed Arc Temp | 3.630 | 0.810 | Inf | 4.503 | **<.0001** |
| Remnant v Disturbed Cla Temp | -2.810 | 0.280 | Inf | -10.214 | **<.0001** |
| Remnant v Disturbed Div Temp | -1.391 | 0.280 | Inf | -5.036 | **<.0001** |
| Remnant v Disturbed Glo Temp | 0.752 | 0.020 | Inf | 31.504 | **<.0001** |
| Remnant v Disturbed Unk Temp | -0.842 | 0.020 | Inf | -35.261 | **<.0001** |
| Remnant v Disturbed Arc Trop | 19.043 | 298.860 | Inf | 0.064 | 0.949 |
| Remnant v Disturbed Cla Trop | 28.635 | 56905.150 | Inf | 0.000 | 1.000 |
| Remnant v Disturbed Div Trop | 25.293 | 19047.750 | Inf | 0.001 | 0.999 |
| Remnant v Disturbed Glo Trop | -0.919 | 0.790 | Inf | -1.158 | 0.247 |
| Remnant v Disturbed Unk Trop | -0.487 | 0.790 | Inf | -0.613 | 0.540 |

**Table S4 | Results from differential abundance analyses of arbuscular mycorrhizal famiies by bioclimatic zone and land use**

Differentially abundant taxa based on land use within sites from (A) Boreal and (B) Tropical bioclimatic zones and (B) across all sites. Only amplicon sequence variants (ASVs) with an adjusted p-value of <0.1 are reported.

| **A. Boreal** | | | | | | | | |
| --- | --- | --- | --- | --- | --- | --- | --- | --- |
| ASV | baseMean | log2FoldChange | lfcSE | stat | pvalue | padj | Remnant | AMF_family |
| 1569 | 31.364 | -6.083 | 0.935 | -6.506 | 0.000 | 0.000 | remnant | Unknown |
| 1674 | 5.000 | -3.293 | 0.881 | -3.738 | 0.000 | 0.056 | remnant | Glomeraceae |
| **B. Tropical** | | | | | | | | |
| ASV | baseMean | log2FoldChange | lfcSE | stat | pvalue | padj | Remnant | AMF_family |
| 1141 | 8.634 | -3.779 | 0.690 | -5.476 | 0.000 | 0.000 | remnant | Unknown |
| 3361 | 4.230 | 3.109 | 0.570 | 5.459 | 0.000 | 0.000 | disturbed | Glomeraceae |
| 7245 | 2.834 | 2.402 | 0.521 | 4.610 | 0.000 | 0.000 | disturbed | Glomeraceae |
| 4576 | 5.015 | -2.860 | 0.653 | -4.381 | 0.000 | 0.000 | remnant | Paraglomeraceae |
| 3580 | 2.534 | 2.188 | 0.541 | 4.041 | 0.000 | 0.002 | disturbed | Glomeraceae |
| 5528 | 2.253 | 1.961 | 0.506 | 3.874 | 0.000 | 0.003 | disturbed | Glomeraceae |
| 11796 | 2.223 | 1.936 | 0.503 | 3.847 | 0.000 | 0.003 | disturbed | Glomeraceae |
| 11962 | 2.201 | 1.916 | 0.502 | 3.817 | 0.000 | 0.003 | disturbed | Unknown |
| 12306 | 2.159 | 1.877 | 0.500 | 3.756 | 0.000 | 0.003 | disturbed | Glomeraceae |
| 3785 | 2.151 | 1.871 | 0.500 | 3.741 | 0.000 | 0.003 | disturbed | Glomeraceae |
| 12868 | 2.091 | 1.812 | 0.497 | 3.650 | 0.000 | 0.004 | disturbed | Glomeraceae |
| 13762 | 2.012 | 1.730 | 0.492 | 3.514 | 0.000 | 0.005 | disturbed | Glomeraceae |
| 3463 | 2.030 | 1.744 | 0.512 | 3.404 | 0.001 | 0.008 | disturbed | Glomeraceae |
| 5930 | 1.858 | 1.562 | 0.479 | 3.259 | 0.001 | 0.012 | disturbed | Glomeraceae |
| 5529 | 1.902 | 1.604 | 0.503 | 3.189 | 0.001 | 0.013 | disturbed | Glomeraceae |
| 9555 | 1.819 | 1.504 | 0.474 | 3.175 | 0.001 | 0.013 | disturbed | Glomeraceae |
| 16696 | 1.800 | 1.489 | 0.471 | 3.159 | 0.002 | 0.013 | disturbed | Glomeraceae |
| 7147 | 1.878 | 1.577 | 0.500 | 3.152 | 0.002 | 0.013 | disturbed | Glomeraceae |
| 5530 | 1.864 | 1.573 | 0.499 | 3.151 | 0.002 | 0.013 | disturbed | Glomeraceae |
| 6901 | 1.858 | 1.553 | 0.498 | 3.119 | 0.002 | 0.014 | disturbed | Glomeraceae |
| 3915 | 1.780 | 1.473 | 0.487 | 3.022 | 0.003 | 0.018 | disturbed | Glomeraceae |
| 19253 | 1.683 | 1.331 | 0.442 | 3.008 | 0.003 | 0.018 | disturbed | Glomeraceae |
| 7959 | 1.697 | 1.365 | 0.472 | 2.892 | 0.004 | 0.024 | disturbed | Glomeraceae |
| 9158 | 1.705 | 1.361 | 0.473 | 2.876 | 0.004 | 0.024 | disturbed | Glomeraceae |
| 9357 | 1.705 | 1.361 | 0.473 | 2.876 | 0.004 | 0.024 | disturbed | Glomeraceae |
| 10016 | 1.660 | 1.300 | 0.464 | 2.802 | 0.005 | 0.029 | disturbed | Glomeraceae |
| 10478 | 1.583 | 1.196 | 0.434 | 2.755 | 0.006 | 0.032 | disturbed | Glomeraceae |
| 8848 | 1.642 | 1.272 | 0.467 | 2.725 | 0.006 | 0.034 | disturbed | Glomeraceae |
| 5502 | 1.593 | 1.218 | 0.449 | 2.712 | 0.007 | 0.034 | disturbed | Glomeraceae |
| 4793 | 1.595 | 1.205 | 0.449 | 2.682 | 0.007 | 0.035 | disturbed | Glomeraceae |
| 7610 | 1.595 | 1.205 | 0.449 | 2.682 | 0.007 | 0.035 | disturbed | Glomeraceae |
| 8749 | 1.575 | 1.173 | 0.444 | 2.639 | 0.008 | 0.039 | disturbed | Glomeraceae |
| 8423 | 2.082 | -1.395 | 0.531 | -2.625 | 0.009 | 0.039 | remnant | Unknown |
| 8035 | 1.530 | 1.122 | 0.434 | 2.584 | 0.010 | 0.043 | disturbed | Glomeraceae |
| 5030 | 1.531 | 1.104 | 0.434 | 2.544 | 0.011 | 0.044 | disturbed | Glomeraceae |
| 9663 | 1.530 | 1.103 | 0.434 | 2.544 | 0.011 | 0.044 | disturbed | Glomeraceae |
| 25280 | 1.478 | 1.027 | 0.404 | 2.543 | 0.011 | 0.044 | disturbed | Glomeraceae |
| 24349 | 1.509 | 1.068 | 0.429 | 2.492 | 0.013 | 0.049 | disturbed | Gigasporaceae |
| 24351 | 1.508 | 1.068 | 0.428 | 2.492 | 0.013 | 0.049 | disturbed | Glomeraceae |
| 26212 | 1.459 | 0.991 | 0.401 | 2.474 | 0.013 | 0.050 | disturbed | Glomeraceae |
| 24353 | 1.489 | 1.052 | 0.448 | 2.350 | 0.019 | 0.062 | disturbed | Glomeraceae |
| 24355 | 1.489 | 1.052 | 0.448 | 2.350 | 0.019 | 0.062 | disturbed | Glomeraceae |
| 24356 | 1.489 | 1.052 | 0.448 | 2.350 | 0.019 | 0.062 | disturbed | Glomeraceae |
| 24357 | 1.489 | 1.052 | 0.448 | 2.350 | 0.019 | 0.062 | disturbed | Glomeraceae |
| 24358 | 1.489 | 1.052 | 0.448 | 2.350 | 0.019 | 0.062 | disturbed | Glomeraceae |
| 28282 | 1.421 | 0.917 | 0.393 | 2.334 | 0.020 | 0.062 | disturbed | Glomeraceae |
| 10628 | 1.487 | 1.032 | 0.446 | 2.312 | 0.021 | 0.062 | disturbed | Unknown |
| 25275 | 1.487 | 1.032 | 0.446 | 2.312 | 0.021 | 0.062 | disturbed | Glomeraceae |
| 25276 | 1.487 | 1.032 | 0.446 | 2.312 | 0.021 | 0.062 | disturbed | Unknown |
| 25278 | 1.486 | 1.031 | 0.446 | 2.311 | 0.021 | 0.062 | disturbed | Glomeraceae |
| 7146 | 1.927 | -1.255 | 0.548 | -2.288 | 0.022 | 0.063 | remnant | Ambisporaceae |
| 25281 | 1.468 | 1.017 | 0.444 | 2.288 | 0.022 | 0.063 | disturbed | Gigasporaceae |
| 26210 | 1.465 | 0.994 | 0.443 | 2.246 | 0.025 | 0.069 | disturbed | Glomeraceae |
| 26214 | 1.447 | 0.980 | 0.441 | 2.223 | 0.026 | 0.070 | disturbed | Glomeraceae |
| 26215 | 1.447 | 0.980 | 0.441 | 2.223 | 0.026 | 0.070 | disturbed | Glomeraceae |
| 26216 | 1.447 | 0.980 | 0.441 | 2.223 | 0.026 | 0.070 | disturbed | Glomeraceae |
| 5053 | 1.861 | -1.191 | 0.540 | -2.204 | 0.028 | 0.070 | remnant | Ambisporaceae |
| 27185 | 1.445 | 0.957 | 0.439 | 2.179 | 0.029 | 0.070 | disturbed | Glomeraceae |
| 27189 | 1.445 | 0.957 | 0.439 | 2.179 | 0.029 | 0.070 | disturbed | Glomeraceae |
| 27226 | 1.444 | 0.956 | 0.439 | 2.177 | 0.029 | 0.070 | disturbed | Gigasporaceae |
| 27223 | 1.443 | 0.955 | 0.439 | 2.176 | 0.030 | 0.070 | disturbed | Glomeraceae |
| 27224 | 1.443 | 0.955 | 0.439 | 2.176 | 0.030 | 0.070 | disturbed | Gigasporaceae |
| 27225 | 1.443 | 0.955 | 0.439 | 2.176 | 0.030 | 0.070 | disturbed | Glomeraceae |
| 27230 | 1.426 | 0.943 | 0.437 | 2.156 | 0.031 | 0.072 | disturbed | Glomeraceae |
| 4949 | 1.794 | -1.123 | 0.530 | -2.120 | 0.034 | 0.075 | remnant | Paraglomeraceae |
| 28239 | 1.423 | 0.918 | 0.435 | 2.107 | 0.035 | 0.075 | disturbed | Glomeraceae |
| 28283 | 1.422 | 0.917 | 0.435 | 2.105 | 0.035 | 0.075 | disturbed | Glomeraceae |
| 12149 | 1.421 | 0.916 | 0.435 | 2.104 | 0.035 | 0.075 | disturbed | Glomeraceae |
| 28281 | 1.421 | 0.916 | 0.435 | 2.104 | 0.035 | 0.075 | disturbed | Glomeraceae |
| 28287 | 1.421 | 0.916 | 0.435 | 2.104 | 0.035 | 0.075 | disturbed | Glomeraceae |
| 12701 | 1.405 | 0.905 | 0.434 | 2.086 | 0.037 | 0.075 | disturbed | Glomeraceae |
| 28291 | 1.405 | 0.905 | 0.434 | 2.086 | 0.037 | 0.075 | disturbed | Glomeraceae |
| 28293 | 1.405 | 0.905 | 0.434 | 2.086 | 0.037 | 0.075 | disturbed | Glomeraceae |
| 2738 | 1.730 | -1.053 | 0.517 | -2.037 | 0.042 | 0.081 | remnant | Ambisporaceae |
| 29406 | 1.402 | 0.877 | 0.432 | 2.032 | 0.042 | 0.081 | disturbed | Glomeraceae |
| 29441 | 1.400 | 0.876 | 0.431 | 2.030 | 0.042 | 0.081 | disturbed | Glomeraceae |
| 29446 | 1.400 | 0.876 | 0.431 | 2.030 | 0.042 | 0.081 | disturbed | Unknown |
| 29447 | 1.399 | 0.875 | 0.431 | 2.029 | 0.042 | 0.081 | disturbed | Glomeraceae |
| 29450 | 1.384 | 0.865 | 0.430 | 2.013 | 0.044 | 0.083 | disturbed | Glomeraceae |
| 30762 | 1.380 | 0.835 | 0.427 | 1.954 | 0.051 | 0.092 | disturbed | Glomeraceae |
| 30828 | 1.377 | 0.834 | 0.427 | 1.951 | 0.051 | 0.092 | disturbed | Glomeraceae |
| 30829 | 1.377 | 0.834 | 0.427 | 1.951 | 0.051 | 0.092 | disturbed | Glomeraceae |
| 30830 | 1.364 | 0.825 | 0.426 | 1.937 | 0.053 | 0.092 | disturbed | Glomeraceae |
| 30831 | 1.364 | 0.825 | 0.426 | 1.937 | 0.053 | 0.092 | disturbed | Glomeraceae |
| 30832 | 1.364 | 0.825 | 0.426 | 1.937 | 0.053 | 0.092 | disturbed | Glomeraceae |
| **C. Across biomes** | | | | | | | | |
| ASV | baseMean | log2FoldChange | lfcSE | stat | pvalue | padj | Remnant | AMF_family |
| 1569 | 9.157 | -4.360 | 0.462 | -9.442 | 0.000 | 0.000 | remnant | Unknown |
| 1674 | 8.582 | -4.272 | 0.510 | -8.373 | 0.000 | 0.000 | remnant | Glomeraceae |
| 1141 | 10.215 | 4.086 | 0.639 | 6.391 | 0.000 | 0.000 | disturbed | Unknown |
| 3361 | 2.803 | 1.758 | 0.502 | 3.501 | 0.000 | 0.005 | disturbed | Glomeraceae |
| 4576 | 3.234 | 1.596 | 0.524 | 3.043 | 0.002 | 0.020 | disturbed | Paraglomeraceae |
| 7245 | 2.038 | 1.383 | 0.466 | 2.970 | 0.003 | 0.021 | disturbed | Glomeraceae |
| 9555 | 1.909 | 1.294 | 0.457 | 2.830 | 0.005 | 0.028 | disturbed | Glomeraceae |
| 7959 | 1.820 | 1.216 | 0.460 | 2.644 | 0.008 | 0.043 | disturbed | Glomeraceae |
| 3580 | 1.859 | 1.229 | 0.476 | 2.582 | 0.010 | 0.046 | disturbed | Glomeraceae |
| 5528 | 1.711 | 1.125 | 0.451 | 2.496 | 0.013 | 0.046 | disturbed | Glomeraceae |
| 11796 | 1.699 | 1.115 | 0.448 | 2.486 | 0.013 | 0.046 | disturbed | Glomeraceae |
| 11962 | 1.687 | 1.104 | 0.447 | 2.469 | 0.014 | 0.046 | disturbed | Unknown |
| 12306 | 1.663 | 1.082 | 0.445 | 2.433 | 0.015 | 0.046 | disturbed | Glomeraceae |
| 3785 | 1.663 | 1.081 | 0.445 | 2.427 | 0.015 | 0.046 | disturbed | Glomeraceae |
| 12701 | 1.639 | 1.058 | 0.443 | 2.390 | 0.017 | 0.046 | disturbed | Glomeraceae |
| 12868 | 1.626 | 1.046 | 0.441 | 2.374 | 0.018 | 0.046 | disturbed | Glomeraceae |
| 13533 | 1.592 | 1.009 | 0.438 | 2.303 | 0.021 | 0.052 | disturbed | Entrophosporaceae |
| 13762 | 1.578 | 0.997 | 0.436 | 2.288 | 0.022 | 0.052 | disturbed | Glomeraceae |
| 3463 | 1.579 | 0.984 | 0.449 | 2.194 | 0.028 | 0.062 | disturbed | Glomeraceae |
| 5930 | 1.493 | 0.900 | 0.426 | 2.113 | 0.035 | 0.073 | disturbed | Glomeraceae |
| 5529 | 1.507 | 0.904 | 0.441 | 2.052 | 0.040 | 0.075 | disturbed | Glomeraceae |
| 5530 | 1.505 | 0.903 | 0.440 | 2.050 | 0.040 | 0.075 | disturbed | Glomeraceae |
| 16696 | 1.457 | 0.855 | 0.421 | 2.030 | 0.042 | 0.075 | disturbed | Glomeraceae |
| 7147 | 1.494 | 0.889 | 0.439 | 2.025 | 0.043 | 0.075 | disturbed | Glomeraceae |
| 6901 | 1.483 | 0.875 | 0.437 | 2.001 | 0.045 | 0.076 | disturbed | Glomeraceae |
| 3915 | 1.457 | 0.844 | 0.434 | 1.946 | 0.052 | 0.082 | disturbed | Glomeraceae |
| 854 | 1.568 | 0.859 | 0.443 | 1.939 | 0.053 | 0.082 | disturbed | Ambisporaceae |
| 19253 | 1.385 | 0.762 | 0.403 | 1.891 | 0.059 | 0.088 | disturbed | Glomeraceae |
| 8423 | 1.605 | 0.795 | 0.433 | 1.837 | 0.066 | 0.095 | disturbed | Unknown |
| 9158 | 1.398 | 0.766 | 0.423 | 1.813 | 0.070 | 0.095 | disturbed | Glomeraceae |
| 9357 | 1.398 | 0.766 | 0.423 | 1.813 | 0.070 | 0.095 | disturbed | Glomeraceae |
